# Maternal Hyperglycemia Disrupts Cerebral Cortex Development Through the Foxp1‐Dll1‐Notch Axis

**DOI:** 10.1002/advs.78305

**Published:** 2026-10-26

**Authors:** Longkai He, Ya Jin, Guosheng Liu, Hao Liu, Xinyi Zhu, Weipeng Xu, Wei Li, Meiyao He, Yao Shen, Bingxiao Li, Xuesong Yang, Xiaoyu Wang, Shasha Han

**Affiliations:** ^1^ Division of Neonatology Department of Pediatrics The First Affiliated Hospital of Jinan University Jinan University Guangzhou China; ^2^ Division of Neonatology and Center for Newborn Care Guangzhou Women and Children’s Medical Center Guangzhou Medical University Guangzhou China; ^3^ Department of Histology and Embryology Key Laboratory for Regenerative Medicine of the Ministry of Education College of Medicine Jinan University Guangzhou China; ^4^ Dongguan Binhaiwan Central Hospital (Dongguan Hospital‐Nanfang Hospital Southern Medical University) Dongguan China

**Keywords:** cortical development, Foxp1, maternal hyperglycemia, neural stem cell, notch signaling

## Abstract

Maternal hyperglycemia is associated with adverse neurodevelopmental outcomes in offspring, but the mechanisms by which it affects fetal brain development remain incompletely understood. Using a mouse model of pregestational diabetes mellitus (PGDM), we found that maternal hyperglycemia was associated with reduced cortical thickness and altered cortical lamination. These abnormalities were accompanied by changes in neural stem cell differentiation and asymmetric division, as well as impaired neuronal migration and dendritic development. Transcriptomic analysis identified the transcription factor Foxp1 as markedly downregulated in the PGDM embryonic cortex. Restoring Foxp1 expression in vivo substantially alleviated the observed cortical abnormalities. Mechanistic experiments showed that Foxp1 represses transcription of the Notch ligand Delta‐like 1 (Dll1) and attenuates Notch signaling. Together, these findings implicate the Foxp1–Dll1–Notch axis as one pathway contributing to disrupted corticogenesis in the PGDM model and provide insight into how an adverse intrauterine metabolic environment affects embryonic brain development.

## Graphical abstract

Maternal hyperglycemia disrupts offspring cortical development through a Foxp1–Dll1–Notch signaling axis. In a PGDM mouse model, intrauterine hyperglycemia decreases cortical thickness and perturbs neuronal layering by downregulating Foxp1, which transcriptionally regulates the Notch ligand Dll1. Remarkably, restoring Foxp1 expression in utero rescues these neurodevelopmental deficits, providing a mechanistic framework for diabetes‐associated brain malformations.

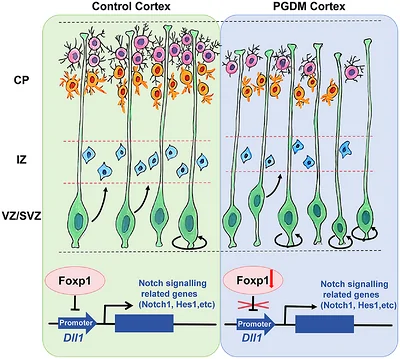

## 1 Introduction

Maternal hyperglycemia is one of the most prevalent complications during pregnancy and is associated with an increased risk of various neurological defects in the developing fetus, including anencephaly, encephalocele, spina bifida [1, 2]. Improvements in prenatal care and glycemic management have substantially reduced the incidence of these severe congenital malformations. Nevertheless, accumulating clinical and experimental evidence suggests that prenatal exposure to maternal hyperglycemia can also affect cerebral cortical development in offspring with an otherwise structurally normal nervous system. Epidemiological studies have further reported an increased incidence of neurodevelopmental and cognitive disorders, including autism spectrum disorder (ASD) and attention‐deficit/hyperactivity disorder (ADHD), among children born to mothers with diabetes [3, 4]. Although these associations are well recognized, the developmental mechanisms by which maternal hyperglycemia influences fetal corticogenesis remain incompletely understood.

The cerebral cortex is generated from neural stem cells (NSCs) residing within the neuroepithelium. Cortical development depends on the coordinated regulation of several NSC populations, including radial glial cells (RGCs), neuroepithelial cells, and basal progenitor cells, with RGCs serving as the principal neural stem cells during corticogenesis [5, 6]. RGCs are highly polarized cells that extend radial processes spanning the developing cortex and generate cortical neurons through precisely regulated symmetric and asymmetric cell divisions. Symmetric divisions expand the progenitor pool by producing two daughter RGCs, whereas asymmetric divisions generate one self‐renewing RGC and one differentiating daughter cell, which may become an intermediate progenitor cell (IPC) or directly differentiate into projection neurons destined for either the deep (layers V‐VI) or upper (layers II‐IV) cortical layers. Newly generated neurons subsequently migrate along radial glial fibers to establish the highly organized laminar architecture of the cerebral cortex [7, 8, 9]. Disruption of these tightly‐regulated processes—including progenitor fate specification, asymmetric division, neuronal differentiation, and migration—can result in abnormal cortical development.

The Forkhead box P1 (Foxp1) is a transcription factor that is abundantly expressed in the developing cerebral cortex and plays an essential role in corticogenesis [10]. Human FOXP1 mutations have been identified in individuals with intellectual disability, language impairment, and autism spectrum disorder, highlighting its importance in normal brain development [10, 11, 12]. Experimental studies have further demonstrated that Foxp1 regulates multiple aspects of cortical development, including radial neuronal migration, NSC differentiation, and dendritic maturation [13, 14]. Mechanistically, Foxp1 promotes neuronal differentiation by modulating Notch signaling through transcriptional repression of Jag1 expression [14]. Despite these advances, whether Foxp1 contributes to cortical developmental abnormalities induced by maternal hyperglycemia, and the underlying molecular mechanisms involved, remain unknown.

In this study, we investigated the effects of maternal hyperglycemia on embryonic cortical development using a pregestational diabetes mellitus (PGDM) mouse model. We examined how intrauterine exposure to elevated glucose levels influences key developmental processes, including neural stem cell asymmetric division, differentiation, migration, and dendritic development, and explored the involvement of Foxp1. Our findings identify a previously unrecognized Foxp1‐Dll1‐Notch signaling axis that mediates cortical developmental abnormalities induced by maternal hyperglycemia, providing mechanistic insight into how an adverse intrauterine metabolic environment may perturb corticogenesis and potentially contribute to altered neurodevelopmental outcomes.

## 2 Results

### 2.1 Maternal Hyperglycemia Disrupts Cortical Lamination During Embryonic Development

Blood glucose levels were monitored as previously described [15], and successful establishment of the PGDM model was confirmed by blood glucose concentrations exceeding 16 mmol L^−1^ (Figure 1b). To examine the effects of maternal hyperglycemia on corticogenesis, embryos were analyzed at E12.5, E15.5, E18.5, and P1, representing key stages of cortical development, including preplate formation, peak neurogenesis accompanied by active neuronal differentiation and migration, cortical lamination, and early postnatal assessment of cortical development [16]. These stages approximately correspond to 8, 20, 30, and 34 weeks of human gestation, respectively [17].

**FIGURE 1:**
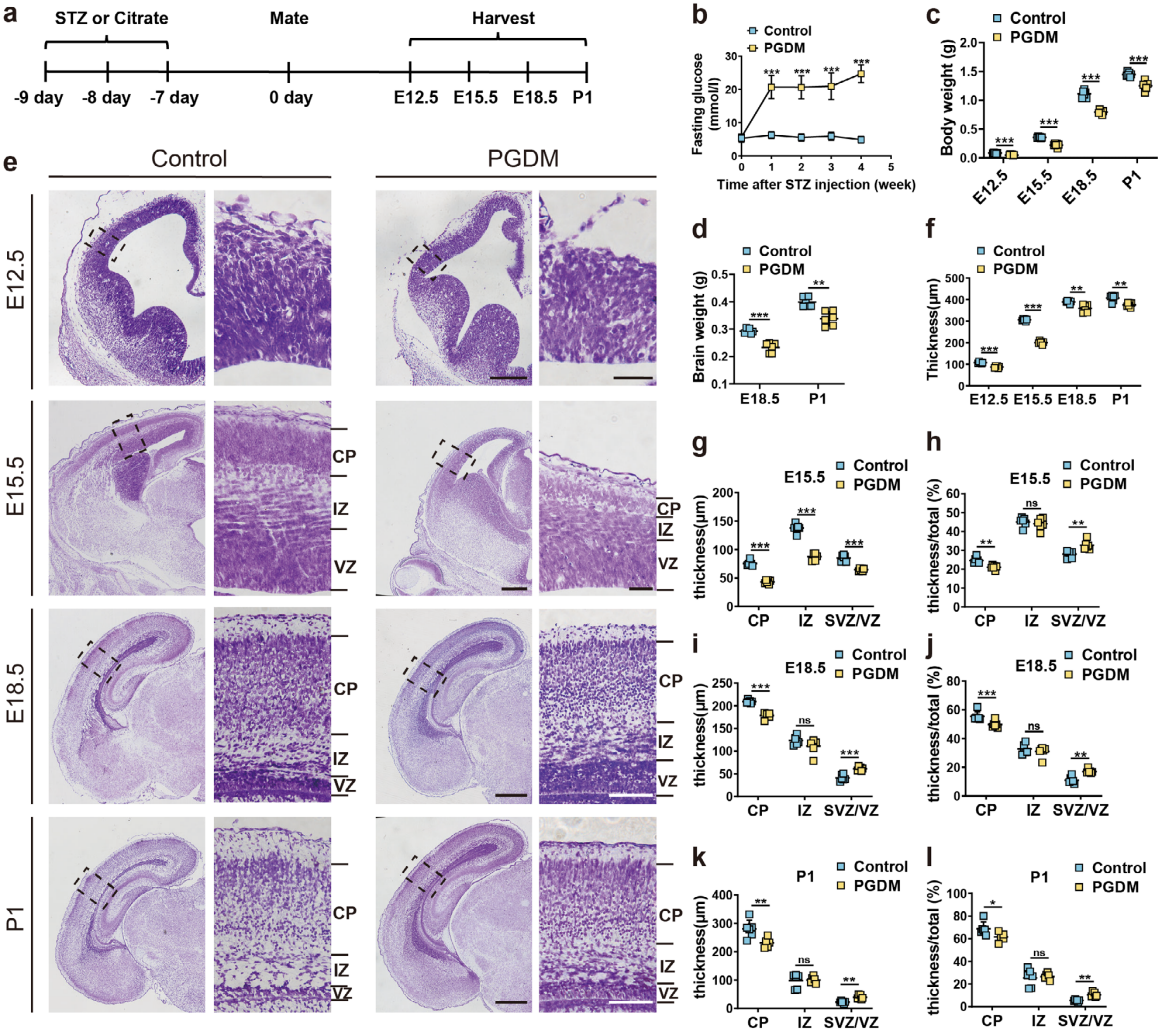
Maternal hyperglycemia disrupts cortical lamination during embryonic development. (a) Experimental timeline illustrating injection, timed mating, and fetus collection. (b) The measurement of maternal blood glucose levels in mice during weeks 0–4, *n* = 10 mice per group. (c) Body weight of embryos at E12.5, E15.5, E18.5, and P1 in the control and PGDM groups. (d) Brain weight in E18.5 and P1 in control and PGDM groups. (e) Representative Nissl‐stained coronal sections of the cerebral cortex from control and PGDM embryos at E12.5, E15.5, E18.5, and P1. (f) Quantification of total cortical thickness in (e). (g, i, k) Quantification of the thickness of the VZ/SVZ, IZ, and CP at E15.5, E18.5, and P1 cortices in (e). (h, j, l) Relative thickness of the VZ/SVZ, IZ, and CP expressed as a percentage of total cortical thickness in (e). For panels (c–l) *n* = 3 independent dams per group (2 embryos per litter). Detailed sample sizes and statistical analyses are provided in Tables S1–S3. Data are presented as mean ± SD; ***p<0.001, **p<0.01 and *p<0.05, ns = not significant. Scale bars: 50 µm in E12.5 and E15.5 (high‐magnification images), 300 µm in E15.5 (low‐magnification images), 400 µm in E18.5 and P1 (low‐magnification images), 100 µm for all other images. Abbreviations: CP, cortical plate; E, embryonic day; IZ, intermediate zone; P, postnatal day; PGDM, pregestational diabetes mellitus; STZ, streptozocin; SVZ, subventricular zone; VZ, ventricular zone.

Compared with control pregnancies, PGDM dams produced significantly fewer embryos at each developmental stage and exhibited a higher incidence of malformed embryos (Figure S1a,b). After excluding malformed embryos, grossly normal PGDM fetuses showed significantly reduced body weight at E12.5, E15.5, E18.5, and P1 (Figure 1c) and reduced brain weight at E18.5 and P1 (Figure 1d). Nissl staining further revealed that the cerebral cortex was consistently thinner in PGDM fetuses than in age‐matched controls throughout the developmental period examined (Figure 1e,f).

To further characterize cortical organization, we quantified the major cortical layers during development. Neural stem cells (NSCs) reside within the ventricular zone (VZ) and subventricular zone (SVZ), where they proliferate and differentiate before newly generated neurons migrate through the intermediate zone (IZ) to populate the cortical plate (CP) [18]. At E15.5, the thickness of the VZ/SVZ was reduced in PGDM embryos compared with controls. In contrast, at E18.5 and P1, the VZ/SVZ became significantly thicker in the PGDM cortex (Figure 1g,i,k). Correspondingly, the proportion of the VZ/SVZ relative to total cortical thickness was significantly increased at these later stages (Figure 1h,j,l). Conversely, both the absolute thickness and the relative proportion of the cortical plate were significantly reduced in PGDM embryos (Figure 1g–l), indicating altered cortical organization.

To determine how these abnormalities evolved during development, we further quantified the VZ/SVZ‐to‐total cortical thickness ratio at E15.5, E18.5, and P1. The difference between the PGDM and control groups progressively increased with developmental age, with the largest difference observed at P1 (Figure S1c). These findings indicate that maternal hyperglycemia disrupts the normal organization of cortical progenitor and neuronal layers during embryonic and early postnatal development, resulting in altered corticogenesis rather than normal cortical maturation.

### 2.2 Maternal Hyperglycemia Disrupts Neural Stem Cell Differentiation, Asymmetric Division, Neuronal Migration, and Dendritic Development During Corticogenesis

The VZ/SVZ primarily comprises Sox2^+^ NSCs and Tbr2^+^ intermediate progenitor cells (IPCs), with IPCs arising from Sox2^+^ progenitors during corticogenesis [19]. To determine the cellular basis underlying the altered cortical lamination observed in PGDM embryos, we performed immunofluorescence staining for Sox2 and Tbr2 at E12.5, E15.5, E18.5, and P1 (Figure 2a). Compared with controls, PGDM embryos had more Sox2^+^ NSCs throughout development (Figure 2b), accompanied by fewer Tbr2^+^ IPCs (Figure 2c), and a lower ratio of Tbr2^+^ to Sox2^+^ cells (Figure 2d). Consistent with these findings, the proportion of the Sox2^+^ domain relative to total cortical thickness progressively increased from E15.5 to P1, with the largest difference observed at P1 (Figure S1d–e). Together with the Nissl staining results, these findings indicate an expansion of the neural progenitor compartment and altered progenitor differentiation during cortical development under maternal hyperglycemia.

**FIGURE 2:**
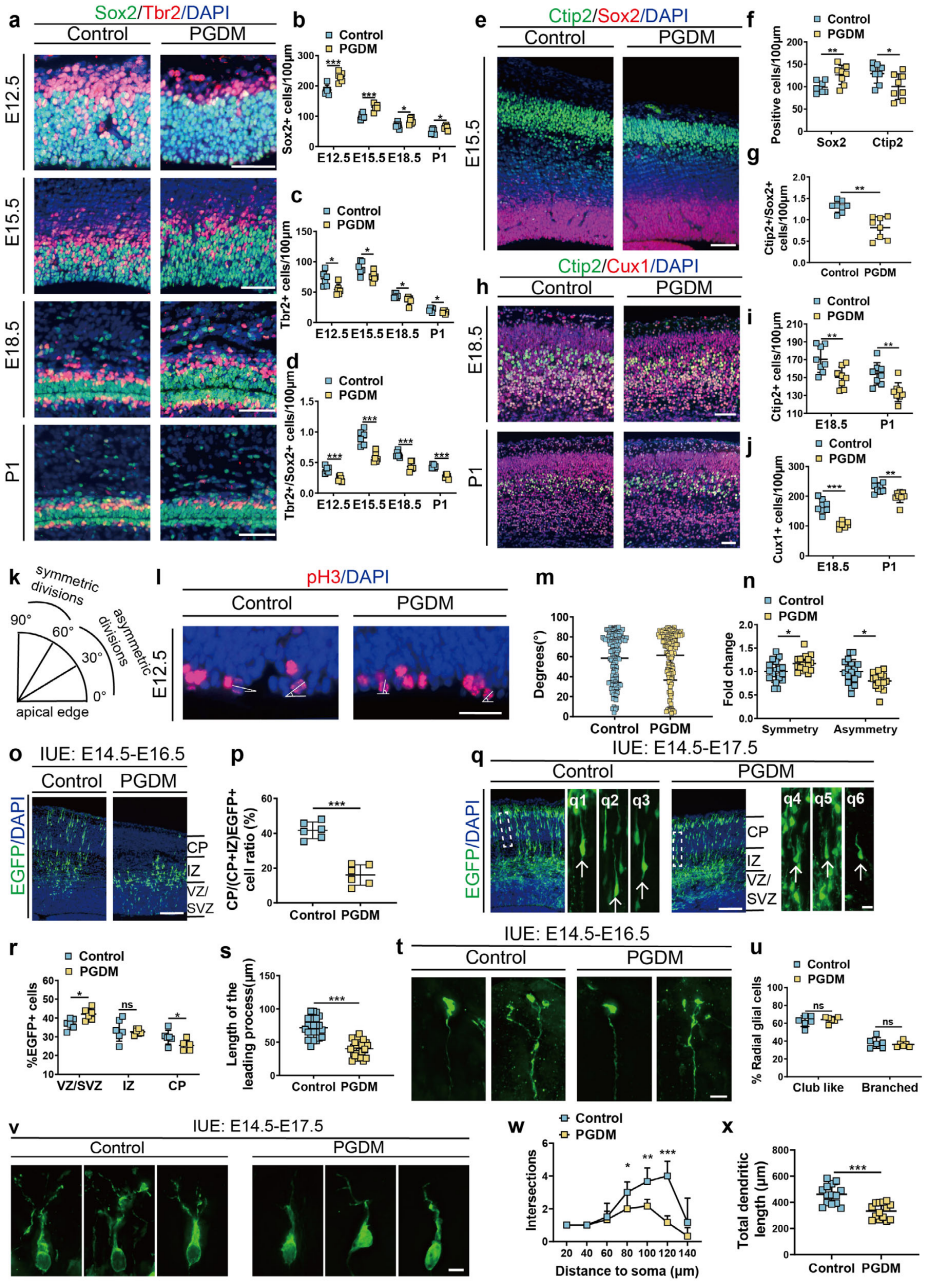
Maternal hyperglycemia impairs neurogenesis and neuronal development in the embryonic cerebral cortex. (a) Representative immunofluorescence images of cortical sections stained for the neural stem cell marker Sox2 (green) and the intermediate progenitor marker Tbr2 (red) at E12.5, E15.5, E18.5, and P1. (b–d) Quantification of Sox2^+^ cells (b), Tbr2^+^ cells (c), and the Sox2/Tbr2 ratio (d), *n* = 3 independent dams per group (2 embryos per litter). (e–g) Representative images of Sox2 (red) and the deep‐layer neuronal marker Ctip2 (green) at E15.5 (e), with quantification of Sox2^+^ cells, Ctip2^+^ cells (f) and the Ctip2/Sox2 ratio (g), *n* = 3 independent dams per group (2 embryos per litter). (h–j) Representative images of the deep‐layer neuronal marker Ctip2 (green) and the upper‐layer neuronal marker Cux1 (red) at E18.5 and P1 (h), with quantification of Ctip2^+^ cells (i) and Cux1^+^ cells (j), *n* = 3 independent dams per group (2–3 embryos per litter). (k–n) Analysis of mitotic spindle orientation at E12.5. Representative images showing spindle angle measurements in phospho‐histone H3 (pH3)‐stained mitotic cells (l), distribution of cleavage angles (m), and quantification of symmetric and asymmetric divisions (n), *n* = 3 independent dams per group (2 embryos per litter). (o) Representative images of EGFP‐labeled migrating neurons at E16.5 by IUE of EGFP at E14.5. (p) Quantification of the percentage of EGFP^+^ cells that migrated into the CP relative to the total number of EGFP^+^ cells located in the IZ and CP, *n* = 3 independent dams per group (2 embryos per litter). (q–s) Analysis of neuronal migration at E17.5. Representative EGFP‐labeled neurons with leading processes (arrows indicated in q1‐6), distribution of EGFP^+^ neurons across cortical regions (r), and quantification of leading process length (s), *n* = 3 independent dams per group (2 embryos per litter, 4–5 cells per embryo). (t, u) Representative images (t) and quantification (u) of RGC endfoot morphology (club‐shaped versus branched), *n* = 3 independent dams per group (2 embryos per litter). (v–x) Representative images of dendritic morphology of cortical plate neurons (v), Sholl analysis of dendritic complexity (w), and quantification of total dendritic length (x), *n* = 3 independent dams per group (2 embryos per litter, 2–3 cells per embryo). Detailed sample sizes and statistical analyses are provided in Table S4. Data are presented as mean ± SD; ***p<0.001, **p<0.01 and *p<0.05, ns = not significant. Scale bars: 50 µm in (a), (e), (h), (o), and (q) (low‐magnification images); 20 µm in (l), (q1–q6), (t), and (v). Abbreviations: CP, cortical plate; E, embryonic day; IUE, in utero electroporation; IZ, intermediate zone; P, postnatal day; PGDM, pregestational diabetes mellitus; RGC, radial glial cell; STZ, streptozocin; SVZ, subventricular zone; VZ, ventricular zone.

To further characterize neuronal differentiation, we examined cortical layer‐specific markers, including Sox2, Pax6, Tbr1 (layer VI), Ctip2 (layers V–VI), and Cux1 (layers II–IV) [20]. At E15.5, the cortical plate (CP) was predominantly composed of Tbr1^+^ and Ctip2^+^ neurons (Figure 2e and Figure S1f,i). Compared with controls, PGDM embryos also had fewer Tbr1^+^ and Ctip2^+^ cells (Figure 2f and Figure S1g,j), lower ratios of Tbr1^+^ or Ctip2^+^ cells to Sox2^+^ cells (Figure 2g and Figure S1h), and fewer Ctip2^+^ cells relative to Pax6^+^ cells (Figure S1k). At E18.5 and P1, the numbers of Ctip2^+^ and Cux1^+^ neurons were also reduced in the PGDM cortex (Figure 2h–j). These observations suggest that maternal hyperglycemia impairs cortical neurogenesis, as reflected by the altered proportions of Sox2^+^, Tbr2^+^, and layer‐specific marker‐positive cells [21].

Neural stem cell fate is closely associated with the orientation of mitotic cleavage. Symmetric divisions (61°–90° relative to the ventricular surface) primarily expand the progenitor pool, whereas asymmetric divisions (0°–60°) generate IPCs and newborn neurons [22, 23]. To determine whether maternal hyperglycemia affects division patterns, mitotic NSCs were labeled with phospho‐histone H3 (pH3) at E12.5 (Figure 2k–l), and division angles were quantified (Figure 2m). PGDM embryos displayed a significant increase in symmetric divisions accompanied by a corresponding reduction in asymmetric divisions (Figure 2n), indicating that maternal hyperglycemia alters the balance between progenitor self‐renewal and differentiation.

To determine whether neuronal migration was also affected, pCIG‐EGFP plasmid was introduced into cortical progenitors at E14.5 by in utero electroporation (IUE), and neuronal distribution was examined at E16.5 (Figure 2o) and E17.5 (Figure 2q). Compared with controls, a greater proportion of EGFP^+^ cells remained within the VZ/SVZ, whereas fewer neurons reached the cortical plate in PGDM embryos (Figure 2p,r), indicating impaired radial migration. Consistent with this finding, migrating neurons in PGDM embryos exhibited shortened leading processes and reduced bipolar morphology (Figure 2q1–6,s). Because radial glial cells (RGCs) provide the scaffold for neuronal migration [23, 24], we further examined RGC morphology by electroporating the radial progenitor‐specific BLBP‐EGFP plasmid at E14.5 and analyzed RGCs at E16.5. Typical RGCs possess a single basal process terminating in either a club‐shaped or branched endfoot at the pial surface (Figure 2t). The proportion of club‐shaped endfeet increases during cortical expansion, while abnormal neuronal migration is associated with increased branching of RGC endfeet [24, 25]. Interestingly, we found no significant difference in endfoot morphology between control and PGDM embryos (Figure 2u), suggesting that the migration defects are primarily associated with altered neuronal morphology rather than gross abnormalities in radial glial organization.

Because cortical thickness also depends on dendritic development [26], we next examined dendritic morphology in neurons transfected with pCIG‐EGFP at E14.5 and analyzed at E17.5 (Figure 2v). Sholl analysis revealed significantly reduced dendritic complexity (Figure 2w) and total dendritic length (Figure 2x) in PGDM embryos, indicating impaired dendritic arborization during cortical development.

Collectively, these results demonstrate that maternal hyperglycemia is associated with coordinated abnormalities in NSC differentiation, asymmetric division, neuronal migration, and dendritic development, which together contribute to disrupted corticogenesis and abnormal cortical organization.

### 2.3 Maternal Hyperglycemia Does Not Alter Neural Stem Cell Proliferation, Cell Cycle Progression, or Apoptosis

To determine whether the altered NSC and neuronal populations observed in PGDM embryos were associated with changes in cell proliferation, cell cycle progression, or apoptosis, we conducted a series of complementary experiments.

First, apoptotic cells were identified by cleaved‐Caspase‐3 immunostaining. No significant difference in the number of apoptotic cells was detected between the PGDM and control groups (Figure S1l,m). We next examined NSC proliferation by co‐immunostaining for pH3 and Sox2 (Figure S1n). Quantification revealed no significant difference in the proportion of proliferating Sox2^+^ NSCs between the two groups (Figure S1q). Likewise, co‐labeling of the proliferation marker Ki67 with the intermediate progenitor marker Tbr2 showed comparable numbers of proliferating Tbr2^+^ IPCs in control and PGDM cortices (Figure S1o,r).

To evaluate cell cycle progression, pregnant mice were injected with EdU at E11.5 or E14.5 to label S‐phase cells, and embryos were collected 24 h later at E12.5 or E15.5. Cell cycle re‐entry was assessed by quantifying the proportion of EdU^+^ cells that remained Ki67^+^ (Figure S1p). The percentage of EdU^+^Ki67^+^ cells did not differ significantly between the PGDM and control groups (Figure S1s), indicating that maternal hyperglycemia does not measurably alter NSC cell cycle progression during the developmental stages examined.

Collectively, these results indicate that maternal hyperglycemia does not significantly affect NSC proliferation, cell cycle progression, or apoptosis. Instead, the altered cortical organization observed in PGDM embryos is associated primarily with abnormalities in NSC differentiation, mitotic division pattern, neuronal migration, and dendritic development.

### 2.4 Foxp1 Is Downregulated in the Developing Cerebral Cortex of PGDM Embryos

To investigate the molecular mechanisms underlying the cortical abnormalities induced by maternal hyperglycemia, we performed RNA sequencing (RNA‐seq) on cerebral cortices isolated from E15.5 PGDM and control embryos. Differential expression analysis identified 3,518 differentially expressed genes (DEGs; FDR < 0.05, |log2FC| > 1; Figure 3a), indicating widespread transcriptional alterations in the developing cortex following intrauterine exposure to maternal hyperglycemia.

**FIGURE 3:**
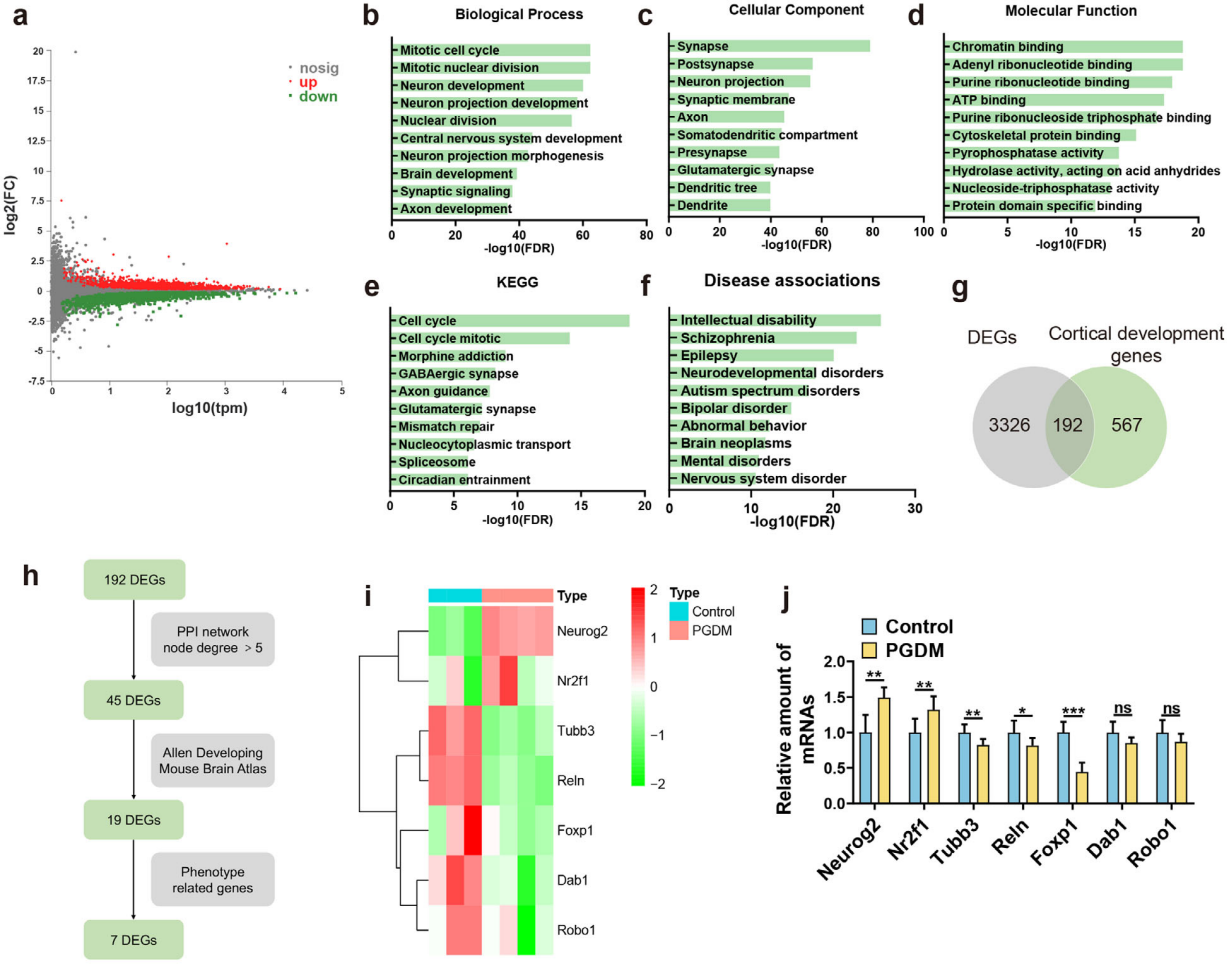
Transcriptomic profiling of E15.5 cerebral cortices identifies dysregulated genes associated with corticogenesis in PGDM embryos. RNA sequencing was performed on cerebral cortices isolated from E15.5 control and PGDM embryos. (a) Volcano plot showing differentially expressed genes (DEGs) between PGDM and control cortices (|log2FC| > 1, FDR < 0.05). (b–d) Gene ontology (GO) enrichment analysis of DEGs: biological process (b), cellular component (c), and molecular functions (d). (e, f) Kyoto Encyclopedia of Genes and Genomes (KEGG) pathway enrichment (e) and Disease Ontology enrichment (f) analyses. The top 10 significantly enriched terms are ranked by ‐log10 (FDR). (g) Venn diagram showing overlap between DEGs and genes associated with cortical development. (h) Workflow illustrating the identification of candidate hub genes. (i) Heatmap showing the expression profiles of the seven dysregulated hub genes identified by transcriptomic analysis (rows: genes; columns: samples). (j) qPCR validation of hub gene expression in E15.5 cerebral cortices from control and PGDM embryos, *n* = 3 independent dams per group (3 embryos per litter). Detailed sample sizes and statistical analyses are provided in Table S5. Data represent mean ± SD; ***p<0.001, **p<0.01, *p<0.05, ns = not significant. Abbreviations: DEGs, differentially expressed genes; KEGG, Kyoto encyclopedia of genes and genomes; PGDM, pregestational diabetes mellitus.

To characterize the biological processes affected, Gene Ontology (GO) enrichment analysis was performed. The DEGs were significantly enriched in pathways related to mitotic nuclear division, neuronal development, neuron projection morphogenesis, chromatin binding, synapse formation, and dendritic development (Figure 3b–d). Kyoto Encyclopedia of Genes and Genomes (KEGG) pathway analysis further revealed enrichment of genes involved in cell cycle regulation, GABAergic and glutamatergic synaptic signaling, axon guidance, and mismatch repair (Figure 3e). Disease enrichment analysis showed that these DEGs were associated with several neurological and neurodevelopment‐related disorders, including intellectual disability and autism spectrum disorder (Figure 3f). Collectively, these analyses indicate that maternal hyperglycemia is associated with broad transcriptional changes affecting biological processes essential for corticogenesis.

Because our RNA‐seq dataset identified thousands of dysregulated genes, we next sought to prioritize candidate regulators that might contribute to the cortical phenotypes observed in PGDM embryos. Intersection analysis identified 192 DEGs associated with cortical development (Figure 3g). Protein‐protein interaction (PPI) network analysis further identified 45 genes with extensive interaction networks (>5 interacting nodes) (Figure 3h). To refine the candidate list, these genes were integrated with expression data from the Allen Brain Atlas and published functional studies of cortical development, allowing us to prioritize genes that are highly expressed in the developing cerebral cortex and have established roles in corticogenesis (Figure 3h). This analysis identified seven candidate genes that were strongly associated with the developmental abnormalities observed in the PGDM cortex (Figure 3i). Quantitative PCR validation demonstrated that Foxp1 exhibited the greatest reduction in expression among these candidates (Figure 3j), identifying Foxp1 as a key transcriptional regulator for subsequent mechanistic investigation.

### 2.5 Foxp1 Is Broadly Expressed in Neural Progenitors and Cortical Neurons and is Downregulated in PGDM Embryos

To characterize the spatiotemporal expression pattern of Foxp1 during cortical development, we examined its distribution in the embryonic cerebral cortex from E12.5 to P1 by immunofluorescence staining (Figure 4a and Figure S2). At E12.5 and E15.5, Foxp1 was broadly expressed throughout the developing cerebral cortex (Figure 4a,b and Figure S2a,b). By E18.5 and P1, Foxp1 expression became increasingly enriched within the cortical plate, particularly in upper‐layer neurons (layers II–IV) (Figure 4a,c,d and Figure S2a,b). Compared with age‐matched controls, Foxp1 immunoreactivity was consistently reduced throughout the cerebral cortex of PGDM embryos at all developmental stages examined (Figure 4b–d).

**FIGURE 4:**
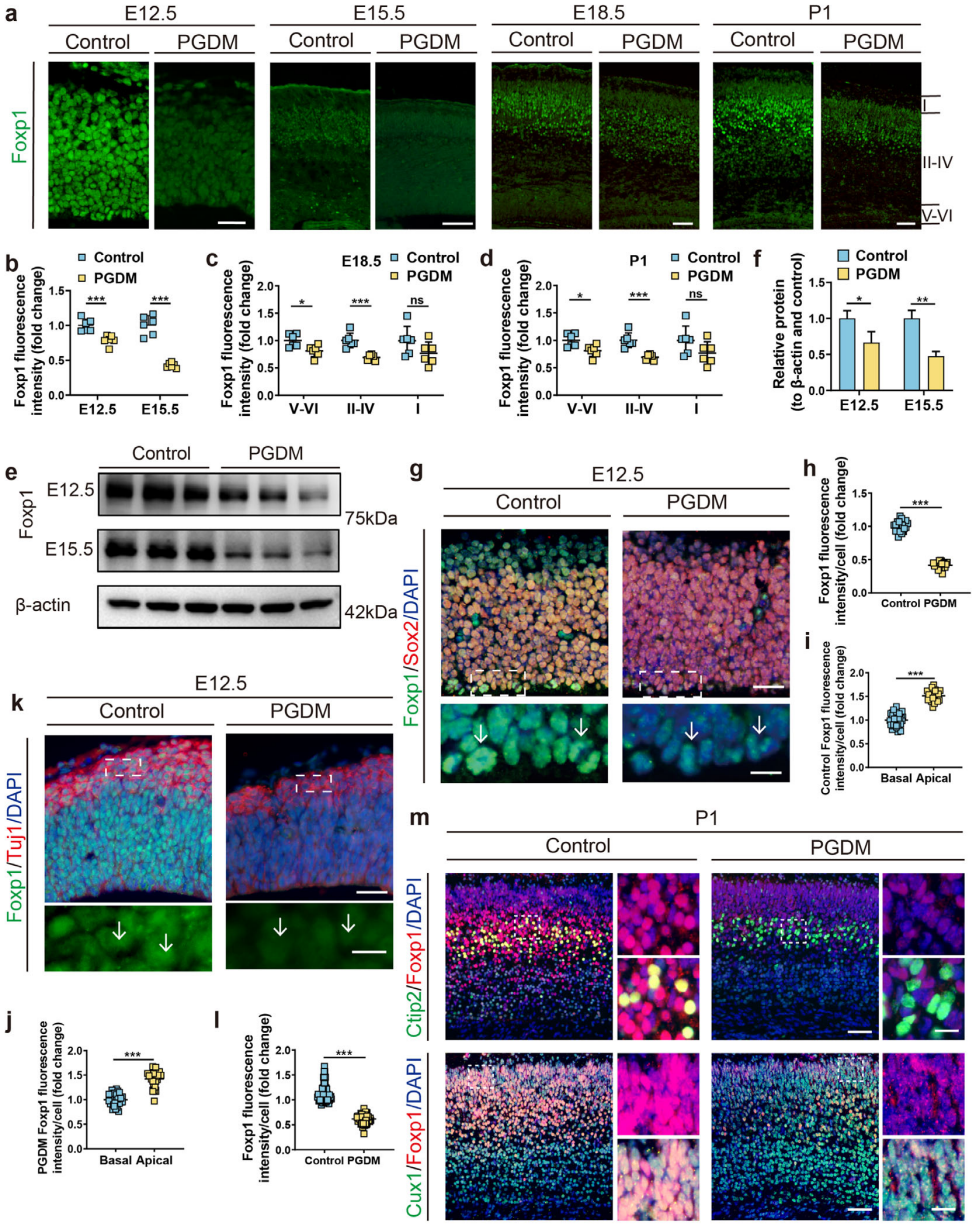
Spatiotemporal expression of Foxp1 in the developing cerebral cortex of control and PGDM embryos. (a) Representative immunofluorescence images showing Foxp1 expression (green) in the VZ and CP at E12.5, E15.5, E18.5, and P1. (b–d) Quantification of normalized Foxp1 immunofluorescence intensity at E12.5, E15.5, E18.5, and P1, *n* = 3 independent dams per group (2 embryos per litter). (e, f) Representative western blots (e) and quantification (f) of Foxp1 protein expression in E12.5 and E15.5 cerebral cortices from control and PGDM embryos, *n* = 3 independent dams per group (1 embryo per litter). (g) Representative images of Sox2 (red) and Foxp1 (green) co‐immunostaining in E12.5 cortices. High‐magnification images (lower panels) show the boxed regions, with white arrows indicating dividing cells (both symmetric and asymmetric divisions). (h, i) Quantification of normalized Foxp1 immunofluorescence intensity in control versus PGDM embryos (h) and apical (dividing) versus basal (non‐dividing) cells (i), *n* = 3 independent dams per group (2 embryos per litter; 3–4 cells per embryo). (j) Comparison of normalized Foxp1 immunofluorescence intensity in dividing cells of PGDM embryos, *n* = 3 independent dams per group (2 embryos per litter; 3–4 cells per embryo). (k) Representative images of Tuj1 (red) and Foxp1 (green) co‐immunostaining in E12.5 cortices. High‐magnification images (lower panels) show the boxed regions, with white arrows indicating intermediate progenitor cells. (l) Quantification of normalized Foxp1 immunofluorescence intensity in intermediate progenitor cells from control and PGDM embryos, *n* = 3 independent dams per group (2 embryos per litter; 10 cells per embryo). (m) Representative images of Ctip2 (upper panels, green) or Cux1 (lower panels, green) co‐immunostained with Foxp1 (red) in P1 cortices. High‐magnification images show boxed regions. Detailed sample sizes and statistical analyses are provided in Table S6. Data are presented as mean ± SD; ***p<0.001, **p<0.01, *p<0.05, ns = not significant. Scale bars: 25 µm in (a) (E12.5); 50 µm in (a) (E15.5, E18.5, and P1), (g), (k), and (m) (low‐magnification images); 20 µm in (g), (k), and (m) (high‐magnification images). Abbreviations: CP, cortical plate; E, embryonic day; P, postnatal day; PGDM, pregestational diabetes mellitus; VZ, ventricular zone.

To validate these observations, we quantified Foxp1 expression by Western blot and quantitative PCR. Both protein (Figure 4e,f) and mRNA (Figure S2c) levels were significantly reduced in the cerebral cortex of PGDM embryos at E12.5 and E15.5, confirming the downregulation of Foxp1 under maternal hyperglycemia. To determine whether Foxp1 expression varies across developmental stages, we further analyzed cortical samples collected at E12.5, E15.5, E18.5, and P1 by Western blot and quantitative PCR. Foxp1 protein and mRNA levels remained relatively stable across these stages (Figure S2d–f), despite the comparatively weaker immunofluorescence signal observed in individual cells at E15.5 (Figure S2a,b).

To identify the cell types expressing Foxp1, we performed double immunostaining with cell type‐specific markers. At E12.5, Foxp1 was highly expressed in Sox2^+^ neural stem cells (Figure 4g), and its immunoreactivity was significantly reduced in Sox2^+^ cells from PGDM embryos (Figure 4h). Foxp1 expression was also detected in pH3‐positive mitotic cells (Figure 4i,j), indicating that Foxp1 is expressed in dividing neural progenitors during corticogenesis. Furthermore, Foxp1 was readily detected in newly differentiated Tuj1^+^ neuronal precursors at E12.5, and its expression was significantly reduced in PGDM embryos (Figure 4k,l). At P1, Foxp1 expression was predominantly localized to cortical neurons, showing strong colocalization with Cux1^+^ upper‐layer neurons and partial colocalization with Ctip2^+^ deep‐layer neurons (Figure 4m). Together, these findings demonstrate that Foxp1 is broadly expressed in neural progenitors and developing cortical neurons during embryonic corticogenesis and is consistently downregulated in the cerebral cortex of PGDM embryos.

### 2.6 Foxp1 Knockdown Disrupts Neural Stem Cell Differentiation and Neuronal Migration During Corticogenesis

To investigate whether reduced Foxp1 expression contributes to the cortical abnormalities observed in PGDM embryos, we knocked down Foxp1 in cortical NSCs by IUE. Prior to in vivo experiments, the knockdown efficiency of the candidate shRNAs was validated in cultured NIH3T3 cells (Figure 5a and Figure S2g,h). The validated pLV‐shFoxp1‐EGFP or control shRNA plasmid was electroporated into the cerebral cortex at E14.5, and brains were collected at E17.5 for analysis (Figure 5b). Both NIH3T3 validation and immunostaining of electroporated cortices confirmed that pLV‐shFoxp1‐EGFP efficiently reduced Foxp1 expression in vitro and in vivo (Figure 5c).

**FIGURE 5:**
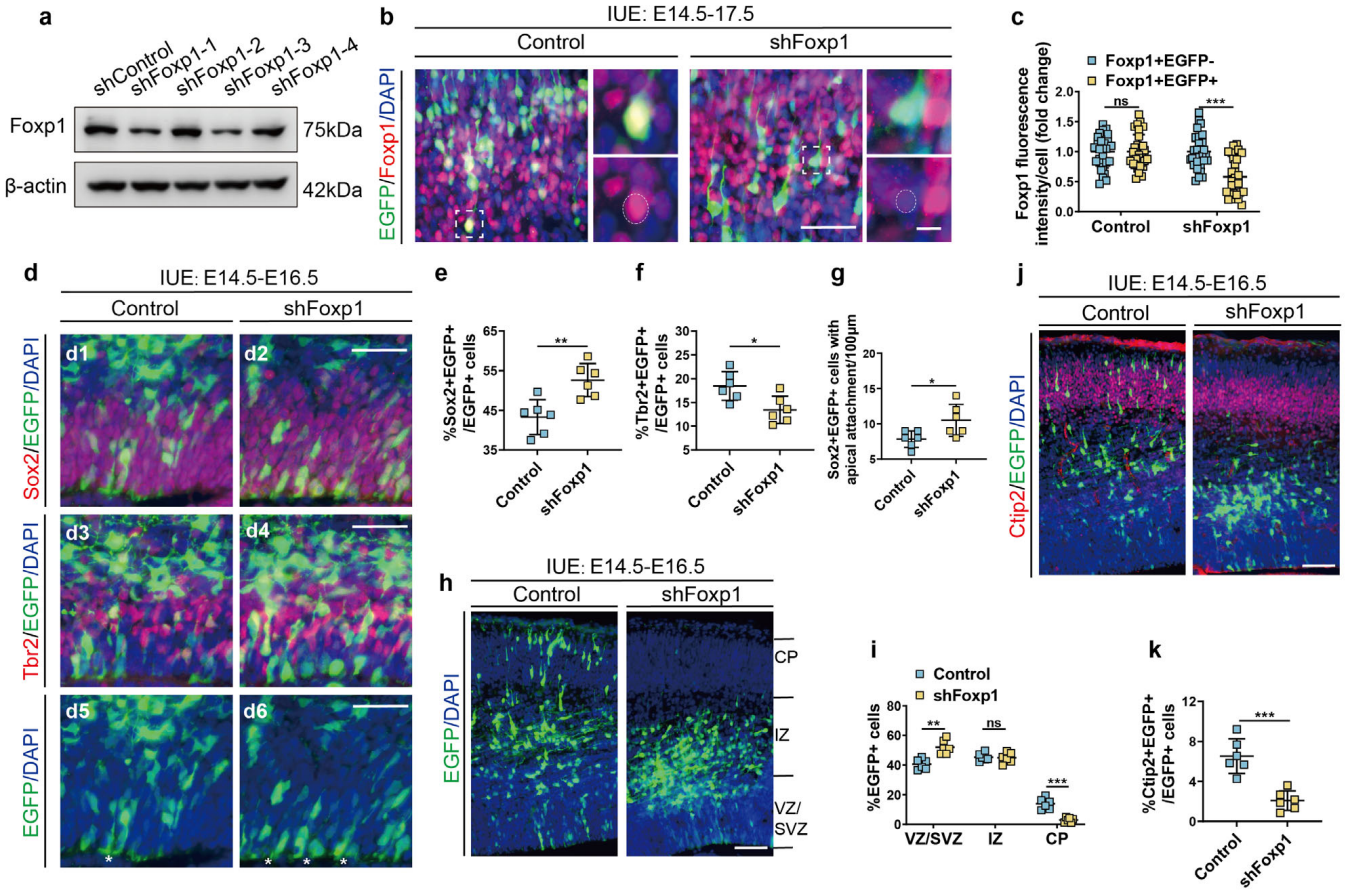
Foxp1 knockdown impairs NSC differentiation and radial migration during cortical development. (a) Representative western blot confirming Foxp1 knockdown following IUE of pLV‐shFoxp1‐EGFP versus control shRNA at E14.5. (b) Representative images of Foxp1 immunostaining (red) in cortical sections after IUE. Higher‐magnification images show the boxed regions, with dashed circles indicating transfected cell nuclei. (c) Quantification of normalized Foxp1 immunofluorescence intensity in control and Foxp1 knockdown embryos, *n* = 3 independent dams per group (1 embryo per litter; 10 cells per embryo). (d) Representative images of EGFP^+^ cells (green) co‐immunostained with Sox2 (upper panels; red) or Tbr2 (middle panels; red) at E16.5. White asterisks indicate apical RGC endfeet contacting the ventricular surface. (e–g) Quantification of the percentage of Sox2^+^EGFP^+^ cells (e), Tbr2^+^EGFP^+^ cells (f), and apical‐attached RGCs (g) among EGFP^+^ cells, *n* = 3 independent dams per group (2 embryos per litter). (h) Representative images showing the distribution of EGFP^+^ cells across cortical layers at E16.5 following IUE. (i) Quantification of the distribution of EGFP^+^ cells within the VZ/SVZ, IZ, and CP, *n* = 3 independent dams per group (2 embryos per litter). (j) Representative images of EGFP^+^ cells co‐immunostained with the deep‐layer neuronal marker Ctip2. (k) Quantification of the percentage of Ctip2^+^EGFP^+^ neurons among EGFP^+^ cells in control and Foxp1 knockdown embryos, *n* = 3 independent dams per group (2 embryos per litter). Detailed sample sizes and statistical analyses are provided in Table S7. Data are presented as mean ± SD; ***p<0.001, **p<0.01, *p<0.05, ns = not significant. Scale bars: 50 µm in (b) (low‐magnification images), (d), (h), and (j); 5 µm in the higher‐magnification images in (b). Abbreviations: CP, cortical plate; E, embryonic day; IUE, in utero electroporation; IZ, intermediate zone; P, postnatal day; PGDM, pregestational diabetes mellitus; RGC, radial glial cell; SVZ, subventricular zone; VZ, ventricular zone.

To determine whether Foxp1 regulates NSC differentiation, we examined the distribution of electroporated EGFP^+^ cells following co‐immunostaining for Sox2 and Tbr2. Compared with controls, Foxp1 knockdown resulted in a greater proportion of EGFP^+^ cells retaining Sox2 expression (Figure 5d1–2,e), accompanied by a significant reduction in EGFP^+^Tbr2^+^ intermediate progenitor cells (Figure 5d3–4,f). These findings indicate that reduced Foxp1 expression is associated with impaired progression of Sox2^+^ NSCs toward the intermediate progenitors.

RGCs, the principal neural stem cells of the developing cortex, undergo progressive morphological changes as corticogenesis proceeds. In the control group, two days after electroporation, many EGFP^+^ RGCs had detached from the ventricular surface and exhibited rounded or elongated morphologies consistent with ongoing differentiation (Figure 5d5). In contrast, Foxp1 knockdown resulted in a larger proportion of EGFP^+^ cells remaining attached to the ventricular surface while maintaining typical radial glial morphology (Figure 5d5,6,g), suggesting delayed morphological progression of RGCs during cortical development.

We next examined the radial distribution of electroporated cells. Foxp1 knockdown resulted in significantly more EGFP^+^ cells remaining within the VZ/SVZ, whereas fewer cells migrated into the CP compared with the control group (Figure 5h,i), indicating impaired radial migration. Consistent with these observations, the number of EGFP^+^Ctip2^+^ deep‐layer neurons was also significantly reduced following Foxp1 knockdown (Figure 5j,k). Together, these findings demonstrate that reduced Foxp1 expression disrupts multiple developmental processes during corticogenesis, including neural stem cell differentiation, radial glial morphological progression, neuronal migration, and the generation of deep‐layer cortical neurons.

### 2.7 Restoration of Foxp1 Expression Substantially Rescues Cortical Developmental Abnormalities in PGDM Embryos

To determine whether restoring Foxp1 expression could rescue the cortical developmental abnormalities induced by maternal hyperglycemia, we constructed a pCIG‐Foxp1 overexpression plasmid and introduced it into cortical NSCs by IUE. Efficient Foxp1 overexpression was confirmed both in cultured NIH3T3 cells (Figure 6b) and in electroporated embryonic cortices by immunostaining (Figure 6a).

**FIGURE 6:**
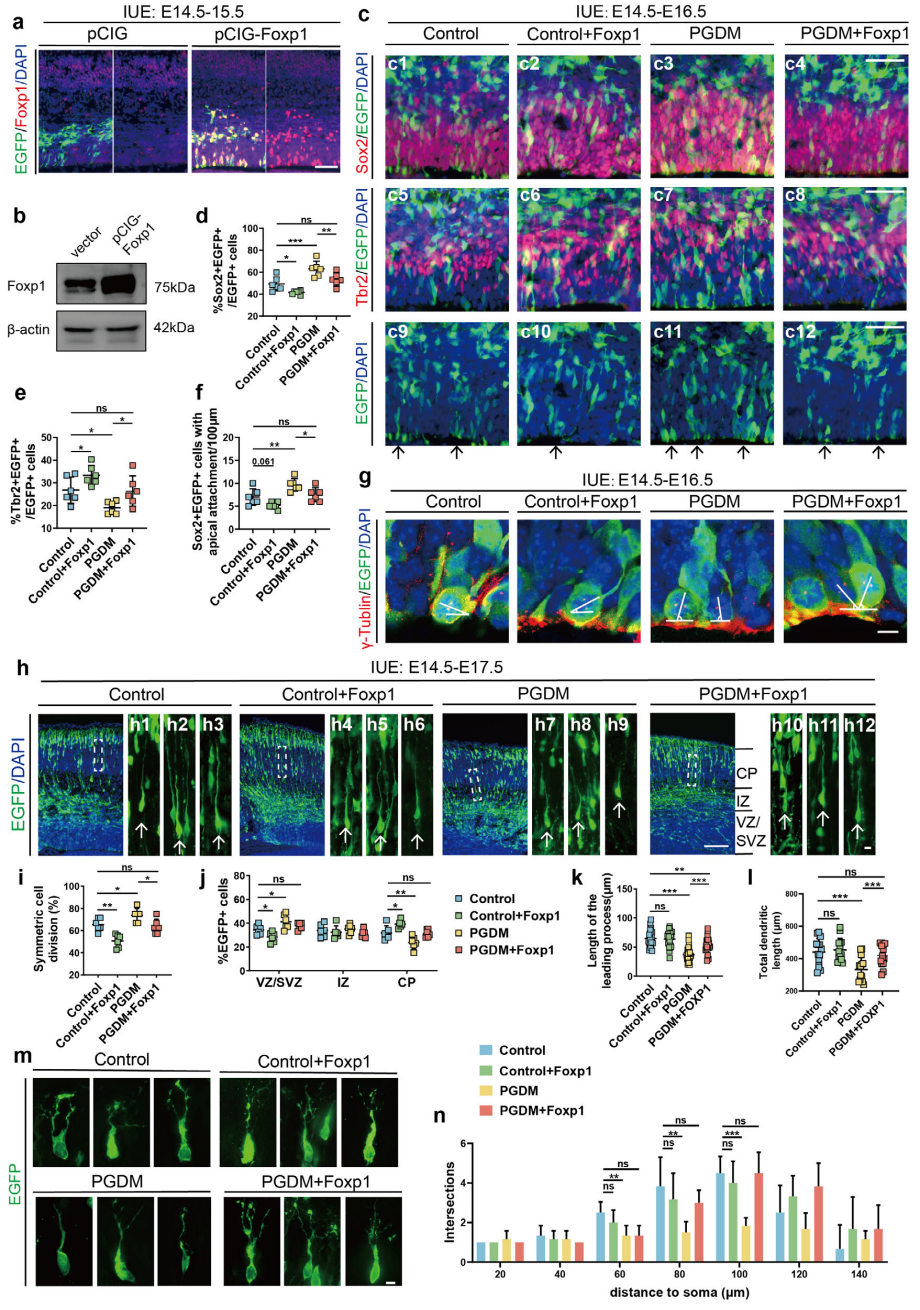
Foxp1 restoration rescues defects in cortical development induced by maternal hyperglycemia. (a) Representative images of Foxp1 overexpression following IUE of pCIG‐Foxp1‐EGFP at E14.5 and immunostaining at E15.5. (b) Representative western blot confirming Foxp1 overexpression, *n* = 3 independent experiments. (c) Representative images of EGFP^+^ cells (green) co‐immunostained with Sox2 (upper panels, red) or Tbr2 (lower panels, red) in the four experimental groups: control + vector, control + Foxp1, PGDM + vector, and PGDM + Foxp1. Black arrows indicate ventricular‐contacting RGCs. (d–f) Quantification of the percentage of Sox2^+^EGFP^+^ cells (d), Tbr2^+^EGFP^+^ cells (e), and apical‐attached RGCs (f), *n* = 3 independent dams per group (2 embryos per litter). (g) Representative images of mitotic spindle orientation analysis via γ‐tubulin (red) and EGFP (green) co‐immunostaining. (h) Representative images of radial migration at E17.5. High‐magnification images (h1–h12) show the boxed regions with representative neurons (white arrows). (i) Quantification of the percentage of symmetric divisions across groups, *n* = 3 independent dams per group (2 embryos per litter). (j) Quantification of the distribution of EGFP^+^ cells within the VZ/SVZ, IZ, and CP, *n* = 3 independent dams per group (2 embryos per litter). (k) Quantification of leading process length, *n* = 3 independent dams per group (2 embryos per litter; 4–5 cells per embryo). (l) Quantification of total dendritic length, *n* = 3 independent dams per group (2 embryos per litter; 4–5 cells per embryo). (m) Representative images of EGFP^+^ neuron morphologies in CP. (n) Sholl analysis of dendritic complexity in EGFP^+^ neurons, *n* = 3 independent dams per group (2 embryos per litter; 2–3 cells per embryo). Detailed sample sizes and statistical analyses are provided in Table S8. Data are presented as mean ± SD; ***p<0.001, **p<0.01, *p<0.05, ns = not significant. Scale bars: 50 µm in (a), (c), and (h); 10 µm in (g), (h1–h12), and (m). Abbreviations: CP, cortical plate; E, embryonic day; IUE, in utero electroporation; IZ, intermediate zone; P, postnatal day; PGDM, pregestational diabetes mellitus; SVZ, subventricular zone; VZ, ventricular zone.

We first examined whether Foxp1 restoration affected NSC differentiation. Compared with the PGDM group, Foxp1 overexpression significantly reduced the proportion of EGFP^+^ cells retaining Sox2 expression, approaching that observed in control embryos (Figure 6c1–4,d). Conversely, the proportion of EGFP^+^Tbr2^+^ intermediate progenitor cells was significantly increased following Foxp1 overexpression (Figure 6c5–8,e). In addition, Foxp1 restoration increased the proportion of RGCs detaching from the ventricular surface, partially restoring their morphological progression toward that observed in the control group (Figure 6c9–12,f).

We next investigated whether Foxp1 influences neural stem cell division patterns. Centrosomes were visualized using γ‐tubulin immunostaining, and cleavage orientation during mitotic anaphase was quantified to distinguish symmetric from asymmetric divisions (Figure 6g) [27, 28]. Foxp1 overexpression significantly increased the proportion of asymmetric divisions in PGDM embryos, shifting the distribution toward that observed in controls (Figure 6g,i). These results indicate that Foxp1 restoration partially normalizes the altered balance between progenitor self‐renewal and differentiation induced by maternal hyperglycemia.

To evaluate neuronal migration, embryos were electroporated at E14.5 and analyzed at E17.5. Foxp1 overexpression significantly increased the proportion of EGFP^+^ cells reaching the cortical plate while reducing the number of cells retained within the VZ/SVZ (Figure 6h,j–l), indicating substantial improvement in radial neuronal migration. Consistent with this observation, the shortened leading processes characteristic of migrating neurons in PGDM embryos were partially restored following Foxp1 overexpression (Figure 6h1–12,k). We further assessed dendritic development in cortical neurons and found that Foxp1 overexpression significantly increased dendritic complexity and total dendritic length compared with the PGDM group, approaching the levels observed in control embryos (Figure 6m,n).

Collectively, these findings demonstrate that restoration of Foxp1 expression substantially rescues multiple developmental abnormalities induced by maternal hyperglycemia, including impaired NSC differentiation, altered mitotic division patterns, defective neuronal migration, and reduced dendritic arborization. These results identify Foxp1 as an important regulator contributing to cortical development under maternal hyperglycemic conditions.

### 2.8 Foxp1 Directly Represses Dll1 Transcription and Modulates Notch Signaling During Cortical Development

To investigate the molecular mechanisms by which Foxp1 regulates cortical development under maternal hyperglycemia, we performed Gene Set Enrichment Analysis (GSEA) using the E15.5 RNA‐seq dataset. GSEA revealed significant enrichment of the Notch signaling pathway in the PGDM cortex (Figure 7a). Consistent with this finding, several Notch ligands, including *Jag2*, *Dll1*, and *Dll3*, were differentially expressed in the PGDM group (Figure 7b).

**FIGURE 7:**
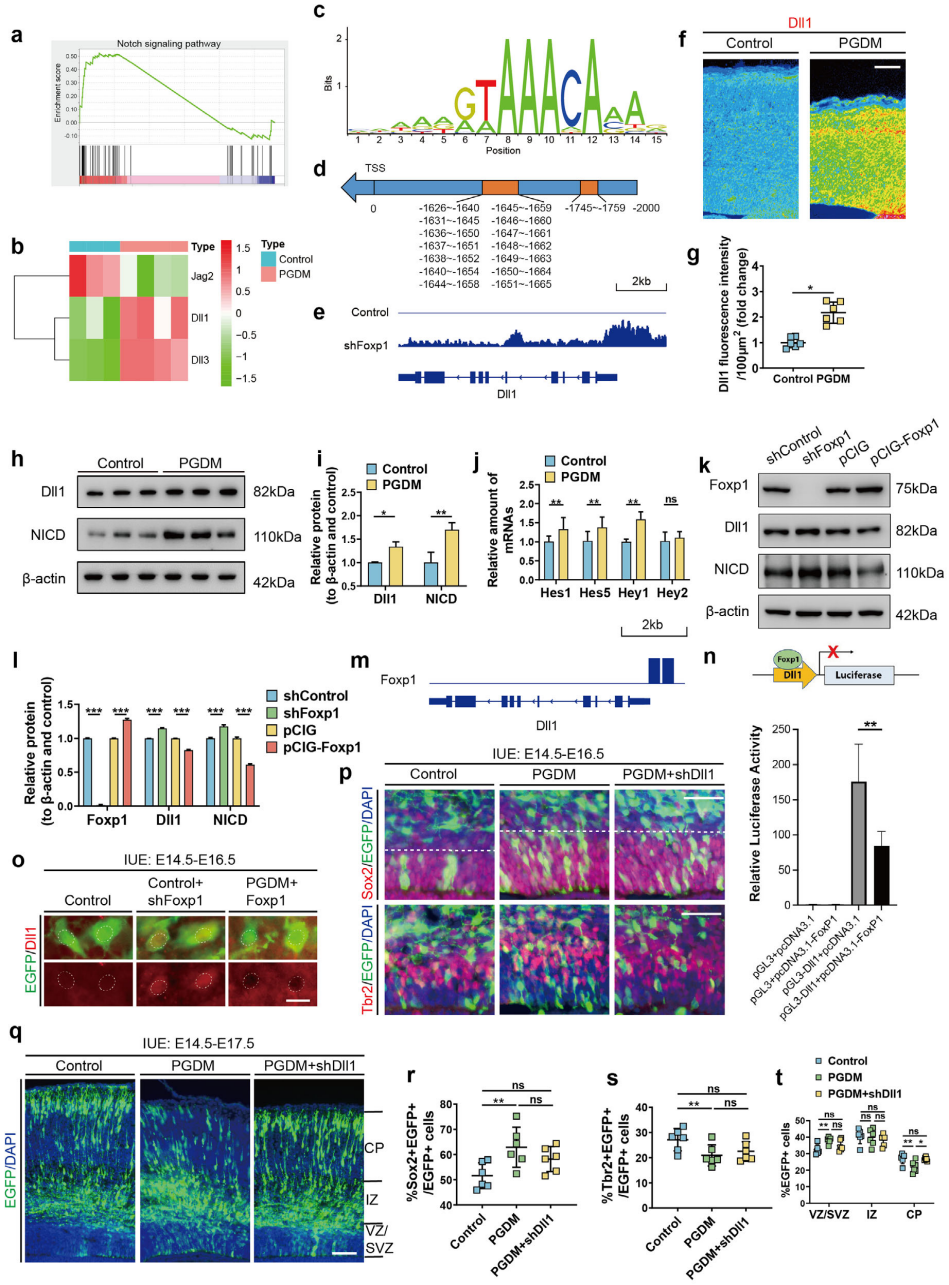
Foxp1 deficiency enhances Dll1 transcription and activates Notch signaling during cortical development. (a) GSEA showing enrichment of Notch signaling pathway in E15.5 PGDM cortices relative to controls. (b) Heatmap showing the expression profiles of differentially expressed Notch ligands. (c) Predicted Foxp1 DNA‐binding motif identified using the JASPAR database. (d) Schematic illustrating the 15 predicted Foxp1‐binding sites within the *Dll1* promoter region relative to the TSS. (e) Genome browser tracks of published Foxp1 ChIP‐seq data showing Foxp1 occupancy at the *Dll1* locus under control and Foxp1 knockdown conditions. (f) Representative images of Dll1 immunostaining in E15.5 cerebral cortices from control and PGDM embryos. (g) Quantification of normalized Dll1 immunofluorescence intensity, *n* = 3 independent dams per group (2 embryos per litter). (h, i) Representative western blots (h) and quantification (i) of Dll1 and NICD protein expression in E12.5 cerebral cortices from control and PGDM embryos, *n* = 3 independent dams per group (1 embryo per litter). (j) qPCR analysis of Notch target genes (*Hes1*, *Hes5*, *Hey1*, and *Hey2*), *n* = 3 independent dams per group (4 embryos per litter). (k, l) Representative western blots (k) and quantification (l) of Dll1 and NICD protein expression following Foxp1 knockdown or overexpression, *n* = 3 independent experiments. (m) CUT&Tag genome browser tracks showing Foxp1 occupancy at the *Dll1* locus in E15.5 control cerebral cortices. (n) Dual‐luciferase reporter assay evaluating the effect of Foxp1 on Dll1 promoter activity, *n* = 3 independent experiments. (o) Representative images of Dll1 immunostaining (red) following IUE. (p) Representative images of EGFP^+^ cells (green) co‐immunostained with Sox2 (upper panels, red) or Tbr2 (lower panels, red) in the three experimental groups: control + vector, PGDM + vector, and PGDM + shDll1. (q) Representative images of EGFP^+^ neurons showing radial migration at E17.5 across groups. (r, s) Quantification of the percentage of Sox2^+^EGFP^+^ cells (r) and Tbr2^+^EGFP^+^ cells (s) among EGFP^+^ cells, *n* = 3 independent dams per group (2 embryos per litter). (t) Quantification of the distribution of EGFP^+^ cells within VZ/SVZ, IZ, and CP, *n* = 3 independent dams per group (2 embryos per litter). Detailed sample sizes and statistical analyses are provided in Tables S9 and S10. Data are presented as mean ± SD; ***p<0.001, **p<0.01, *p<0.05, ns = not significant. Scale bars: 50 µm in (f), and (q); 25 µm in (p); 10 µm in (o). Abbreviations: CP, cortical plate; E, embryonic day; GSEA, Gene Set Enrichment Analysis; IUE, in utero electroporation; IZ, intermediate zone; NICD, Notch intracellular domain; PGDM, pregestational diabetes mellitus; SVZ, subventricular zone; TSS, transcription start site; VZ, ventricular zone.

To identify potential downstream targets of Foxp1 within the Notch signaling pathway, we searched for Foxp1‐binding motifs in the promoter regions of these candidate ligands using the JASPAR database. Bioinformatics analysis identified 15 putative Foxp1‐binding sites within the *Dll1* promoter, whereas no predicted binding sites were detected in the promoter regions of *Jag2* or *Dll3* (Figure 7c,d). Analysis of published Foxp1 ChIP‐seq datasets [14] further revealed enrichment of Foxp1 binding at the *Dll1* promoter (Figure 7e), suggesting that *Dll1* may represent a direct transcriptional target of Foxp1.

We next examined Dll1 expression in the developing cerebral cortex. Immunofluorescence staining demonstrated widespread Dll1 expression throughout the embryonic cortex at E15.5 (Figure 7f), with significantly greater fluorescence intensity in PGDM embryos than in controls (Figure 7g). Western blot and quantitative PCR further confirmed increased Dll1 expression together with elevated levels of the activated Notch signaling component NICD in the PGDM cortex (Figure 7h,i, and Figure S3a). Moreover, expression of the canonical Notch target genes Hes1, Hes5, and Hey1 was significantly increased (Figure 7j). Consistent with these molecular changes, immunostaining revealed significantly stronger Notch1 and Hes1 immunoreactivity at E15.5 and E18.5 in PGDM embryos (Figure S3c–f), indicating increased Notch signaling activity under maternal hyperglycemia.

To investigate the regulatory relationship between Foxp1 and Dll1, we manipulated Foxp1 expression in NIH3T3 cells. Foxp1 knockdown increased Dll1 and NICD expression, whereas Foxp1 overexpression produced the opposite effect (Figure 7k,l and Figure S3b), supporting an inverse relationship between Foxp1 expression and Notch signaling activity. Dual‐luciferase reporter assays further demonstrated that Foxp1 significantly repressed *Dll1* promoter activity (Figure 7n). To determine whether Foxp1 directly binds the *Dll1* promoter in vivo, we performed a CUT&Tag assay using embryonic cortical tissue at E15.5. A marked enrichment of Foxp1 occupancy was detected at the *Dll1* promoter compared with the IgG control (Figure 7m), providing direct evidence that Foxp1 binds the *Dll1* regulatory region during corticogenesis.

To determine whether altered Dll1 expression contributes to the cortical abnormalities associated with Foxp1 deficiency, we knocked down Dll1 in cortical NSCs using IUE. The efficiency of the shRNA constructs was first validated in NIH3T3 cells (Figure S3g–i). Consistent with the in vitro results, Foxp1 knockdown increased Dll1 expression in electroporated cortical cells, whereas Foxp1 overexpression significantly reduced Dll1 expression in vivo (Figure 7o and Figure S3j). We next examined whether reducing Dll1 expression could ameliorate the developmental abnormalities observed in PGDM embryos. Dll1 knockdown decreased the proportion of EGFP^+^Sox2^+^ NSCs while increasing the proportion of EGFP^+^Tbr2^+^ intermediate progenitor cells (Figure 7p,r,s). In addition, Dll1 knockdown increased the proportion of EGFP^+^ cells reaching the cortical plate, indicating substantial improvement in radial neuronal migration (Figure 7q,t).

Collectively, these findings support a model in which reduced Foxp1 expression contributes to increased Dll1 expression and enhanced Notch signaling activity during cortical development. Although additional dysregulated pathways are likely involved in the PGDM cortex, our data identify the Foxp1–Dll1–Notch signaling axis as an important mechanism contributing to the cortical developmental abnormalities induced by maternal hyperglycemia.

## 3 Discussion

The present study investigated the effects of maternal hyperglycemia on embryonic cortical development and examined the role of Foxp1 in regulating neural stem cell fate in a PGDM mouse model. Our findings demonstrate that maternal hyperglycemia is associated with multiple abnormalities in corticogenesis, including impaired asymmetric division of NSCs, reduced neuronal differentiation, defective radial migration, and compromised dendritic development. We further identify a previously unrecognized Foxp1–Dll1–Notch signaling axis that contributes to these developmental abnormalities. Specifically, we show that maternal hyperglycemia is accompanied by reduced Foxp1 expression in the developing cortex, that restoration of Foxp1 substantially rescues cortical developmental defects, and that Foxp1 directly represses *Dll1* transcription, thereby modulating Notch signaling during corticogenesis (Figure 8). Together, these findings provide mechanistic insight into how an adverse intrauterine metabolic environment perturbs cortical development.

**FIGURE 8:**
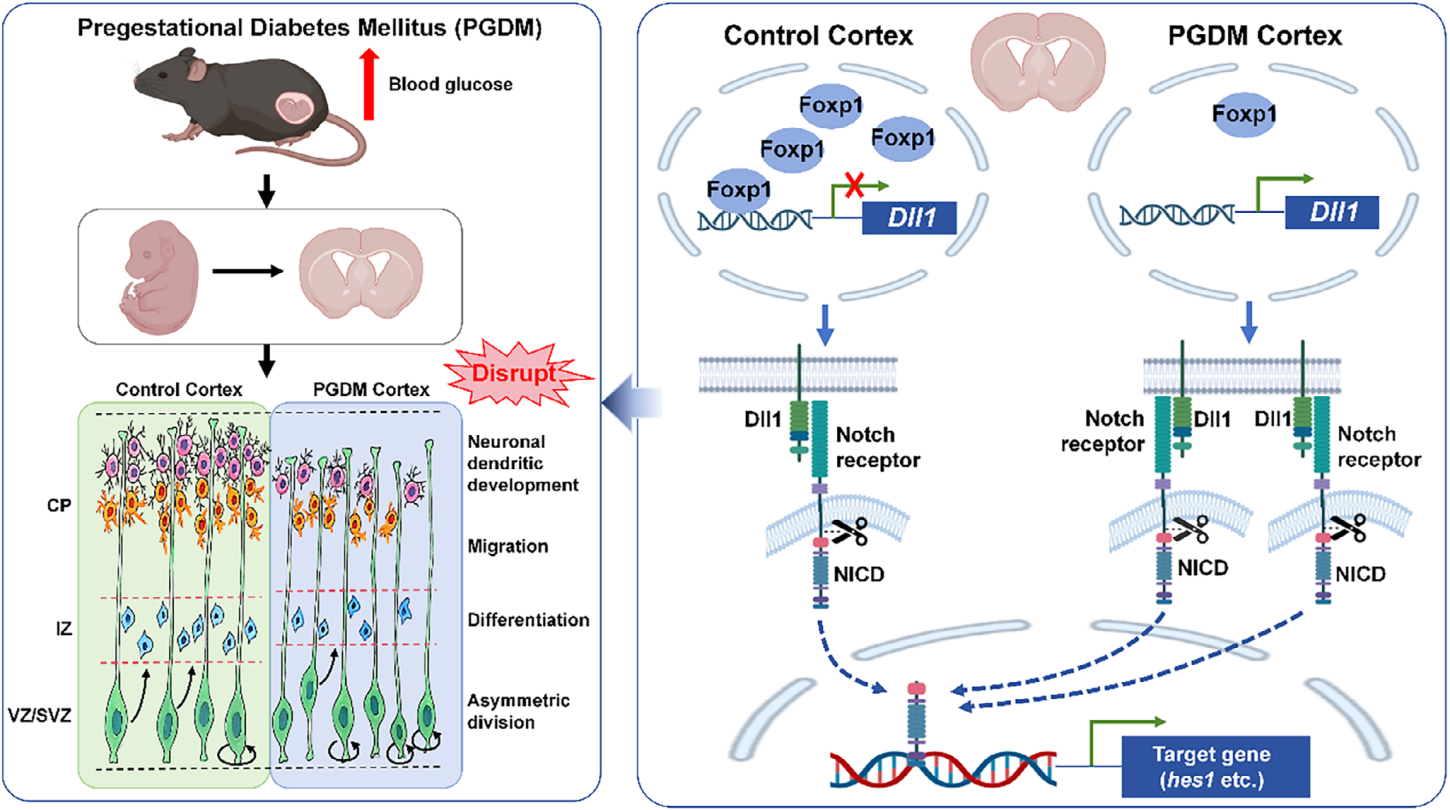
Schematic model of maternal hyperglycemia disrupting cerebral cortex development through the Foxp1‐Dll1‐Notch axis.

Maternal hyperglycemia perturbs cortical development through multiple interconnected developmental processes that regulate NSC fate, neuronal differentiation, and neuronal migration. During corticogenesis, cortical projection neurons are generated through two principal developmental routes. In one pathway, NSCs first generate intermediate progenitor cells (IPCs), which subsequently undergo an additional round of division to produce neurons. Alternatively, NSCs can directly generate postmitotic projection neurons within the VZ without passing through the IPC stage [29]. Previous lineage analyses have further demonstrated that individual IPCs are generally fate‐restricted and typically generate only a limited number of closely related projection neurons, whereas direct neurogenesis from NSCs contributes substantially to neuronal production during early corticogenesis [30].

Our findings suggest that maternal hyperglycemia is associated with alterations in several processes involved in corticogenesis. At the stages examined, PGDM embryos showed a lower proportion of divisions classified as asymmetric, more Sox2^+^ progenitors, fewer Tbr2^+^ intermediate progenitors, and a lower ratio of Tbr2^+^ to Sox2^+^ cells. The numbers of Tbr1^+^, Ctip2^+^, and Cux1^+^ neurons were also reduced. Together, these observations indicate changes in cortical cell populations during development. Because marker counts and ratios do not establish how individual cells progress through a lineage, direct lineage tracing would be needed to determine whether maternal hyperglycemia alters progenitor fate or neuronal differentiation.

Our observations are consistent with an increasing body of literature demonstrating that maternal diabetes disrupts corticogenesis through multiple mechanisms. Transcriptomic analyses of diabetic rat embryos have revealed widespread alterations in genes regulating mitosis and neural development, accompanied by abnormal spindle organization and altered progenitor division patterns [31, 32, 33]. Histological analyses further demonstrated disrupted cortical cytoarchitecture, impaired neuronal migration and polarity, and reduced excitability of deep‐layer projection neurons in offspring exposed to maternal diabetes [34]. More recently, impaired Reelin signaling has been identified as an important contributor to migration defects in diabetic embryopathy [35]. Together with these previous studies, our findings support the concept that maternal hyperglycemia perturbs corticogenesis through coordinated disruption of multiple developmental processes rather than a single cellular event. Our work further extends these observations by identifying Foxp1‐mediated transcriptional regulation as one molecular mechanism contributing to these developmental abnormalities.

In addition to altered progenitor differentiation, previous studies have shown that maternal hyperglycemia affects signaling pathways essential for cortical development. For example, inhibition of ciliary neurotrophic factor (CNTF) signaling in PGDM fetal brains promotes a premature shift from neurogenesis toward gliogenesis during mid‐gestation [15, 34]. Such developmental alterations, together with the changes in progenitor dynamics observed in the present study, are likely to cooperatively influence cortical neuron production. Consistent with this interpretation, our data demonstrate abnormalities in NSC differentiation, mitotic cleavage orientation, and neuronal migration, suggesting that the reduced cortical plate thickness observed in PGDM embryos reflects the combined effects of multiple dysregulated developmental processes rather than any single cellular mechanism.

Impaired neuronal migration appears to represent another important contributor to the cortical abnormalities observed in PGDM embryos. Our in utero electroporation experiments revealed significantly fewer EGFP‐labeled neurons reaching the cortical plate, accompanied by shortened leading processes in migrating neurons. In contrast, radial glial scaffold morphology remained largely preserved, suggesting that defective neuronal morphology, rather than gross abnormalities of radial glial organization, primarily underlies the migration defects observed in the present study. These findings are also consistent with previous reports demonstrating abnormal neuronal polarity and migration in diabetic embryopathy [36, 37].

Maternal hyperglycemia also affected later stages of neuronal development. In the present study, upper‐layer cortical neurons exhibited significantly reduced dendritic complexity and total dendritic length, indicating impaired dendritic arborization. Similar abnormalities have been reported in rat models of gestational diabetes, in which cortical neurons display delayed dendritic maturation together with reduced expression of synaptic proteins, including synaptophysin [36]. Hyperglycemia has also been shown to impair thalamocortical axon elongation during fetal development [37], while reduced glutamate and GABA levels have been reported in embryonic brains of Akita mice [38]. Collectively, these observations suggest that maternal hyperglycemia influences multiple aspects of neuronal maturation, including dendritic development, axonal growth, and synaptic organization, which may further compromise cortical circuit assembly.

Transcriptomic profiling identified Foxp1 as one of the most significantly downregulated transcriptional regulators in the developing cerebral cortex of PGDM embryos. Reduced Foxp1 expression was consistently confirmed by immunohistochemistry, Western blot, and quantitative PCR across multiple developmental stages. Functional studies further demonstrated that Foxp1 knockdown in the developing cortex recapitulated several key phenotypes observed in PGDM embryos, including altered NSC differentiation, impaired neuronal migration, and abnormal cortical organization. Consistent with these findings, deletion of Foxp1 in mouse cortex resulted in overall cortical thinning, disrupted cortical lamination, and altered proportion of cortical layers [39]. Conversely, our restoration of Foxp1 expression substantially rescued these developmental abnormalities. Together, these findings identify Foxp1 as an important regulator contributing to cortical developmental abnormalities associated with maternal hyperglycemia.

Foxp1 plays an essential role during corticogenesis. In line with our observations, Foxp1 deficiency has been shown to increase symmetric division of radial glial cells and impair neuronal radial migration, leading to progenitor cell accumulation in the VZ/SVZ and a concomitant reduction in the cortical plate [13, 40, 41, 42]. Notably, the aberrant positioning of Foxp1‐deficient neurons persists until P14, indicating a long‐lasting developmental disruption [13]. These findings, including abnormal cortical lamination, impaired radial neuronal migration, and altered cortical layer composition, closely resemble the developmental abnormalities observed in the present study, supporting the notion that reduced Foxp1 expression contributes to disrupted corticogenesis under maternal hyperglycemia. Nevertheless, because our transcriptomic analysis identified more than 3500 differentially expressed genes, Foxp1 is unlikely to represent the sole mediator of the PGDM phenotype. Rather, our findings suggest that Foxp1 functions as one important component of a broader transcriptional network regulating cortical development.

Foxp1 regulates NSC differentiation through multiple downstream pathways. Previous studies have demonstrated that Foxp1 promotes neuronal differentiation by repressing Jag1‐dependent Notch signaling in NSCs [14]. In the present study, however, Jag1 expression was not significantly altered in the PGDM cortex, whereas Dll1 was markedly upregulated and exhibited multiple predicted Foxp1‐binding sites within its promoter. CUT&Tag, luciferase reporter assays, and gain‐ and loss‐of‐function experiments consistently demonstrated that Foxp1 directly represses Dll1 transcription, resulting in reduced Notch signaling activity. These findings identify Dll1 as a previously unrecognized downstream target of Foxp1 during cortical development. At the same time, our RNA‐seq analysis also revealed altered expression of additional Notch ligands, including Jag2 and Dll3, indicating that maternal hyperglycemia likely perturbs Notch signaling more broadly. Additionally, increased levels of the NICD protein, and elevated mRNA levels of downstream Notch target genes (*Hes1*, *Hes5*, *Hey1*, and *Hey2*) were detected, further confirming the activation of Notch signaling. Given the diverse and context‐dependent functions of Notch ligands during corticogenesis, future studies will be required to determine the relative contributions of individual ligands to the developmental abnormalities observed in PGDM embryos.

Our transcriptomic data also identified changes in additional regulators of cortical development, including *Pax6* and its downstream target Ngn2, both of which participate in transcriptional networks governing NSC differentiation and neuronal specification. Because Ngn2 can promote Dll1 expression [43], interactions between Foxp1 and other developmental regulators may further influence cortical development under maternal hyperglycemia. In addition, Foxp1 is known to form both homodimers and heterodimers with other FOXP family members, including Foxp2 and Foxp4, thereby modulating transcriptional activity during corticogenesis. Whether maternal hyperglycemia alters these broader FOXP‐dependent transcriptional networks remains unknown and represents an important direction for future investigation.

Beyond its role in progenitor differentiation, Foxp1 also contributes to later stages of neuronal maturation. Restoration of Foxp1 expression significantly improved dendritic complexity and dendritic length in PGDM embryos, consistent with previous studies demonstrating that Foxp1 deficiency impairs dendritic arborization in cultured cortical neurons and in developing cortical projection neurons in vivo [13, 41, 44]. Together with our migration analyses, these findings suggest the long‐term role of Foxp1 in neuronal maturation, spanning from embryonic to postnatal development.

FOXP1 has been firmly established as an essential regulator of human brain development, and pathogenic FOXP1 variants cause a syndromic neurodevelopmental disorder characterized by intellectual disability, language impairment, and autistic features [45, 46]. In addition, multi‐omics studies have shown that Foxp1 regulates transcriptional programs enriched for cortical development, synaptic function, and genes associated with neurodevelopmental disorders [40, 47, 48]. Epidemiological studies have also reported associations between maternal hyperglycemia and an increased risk of adverse neurodevelopmental outcomes in offspring [49, 50, 51]. However, our study examined embryonic cortical development and did not assess postnatal neural circuit function, electrophysiological properties, or behavioral outcomes. Therefore, the relationship between the cortical developmental abnormalities identified here and later neurodevelopmental disorders should be interpreted with caution. Rather than establishing a direct link to neurodevelopmental disorders, our findings provide a molecular framework for understanding how maternal hyperglycemia may perturb embryonic corticogenesis through the Foxp1–Dll1–Notch signaling axis.

Several questions remain to be addressed. The upstream mechanism by which maternal hyperglycemia suppresses Foxp1 expression is still unknown. Moreover, although restoration of Foxp1 substantially rescued the cortical defects induced by maternal hyperglycemia, our transcriptomic analysis indicates that additional dysregulated pathways are also likely to contribute to abnormal corticogenesis. Defining the upstream regulation of Foxp1 and its interaction with other pathogenic pathways will further improve our understanding of the molecular mechanisms underlying cortical developmental abnormalities associated with maternal hyperglycemia.

## 4 Conclusions

In summary, maternal hyperglycemia alters multiple aspects of embryonic corticogenesis, including neural stem cell differentiation, asymmetric division, neuronal migration, and dendritic development. Our findings implicate reduced Foxp1 expression in these abnormalities and show that Foxp1 represses Dll1 transcription and modulates Notch signaling. Restoring Foxp1 expression substantially ameliorated the cortical abnormalities induced by maternal hyperglycemia, supporting the Foxp1–Dll1–Notch axis as one pathway contributing to altered corticogenesis in PGDM embryos. The mechanism by which maternal hyperglycemia reduces Foxp1 expression remains unknown. These findings provide mechanistic insight into the effects of maternal hyperglycemia on fetal brain development while identifying an important question for further investigation.

## 5 Materials and Methods

### 5.1 Experimental Design

This study aimed to elucidate the role of Foxp1 in the cortical developmental abnormalities induced by maternal hyperglycemia. Using a pregestational diabetes mellitus (PGDM) mouse model, we systematically examined key developmental processes during corticogenesis, including neural stem cell differentiation, asymmetric division, neuronal migration, and dendritic development. RNA sequencing was performed to identify transcriptional changes associated with maternal hyperglycemia, followed by functional gain‐ and loss‐of‐function studies to investigate the role of the Foxp1‐Dll1‐Notch signaling axis in vitro and in vivo. Animals were randomly assigned to experimental groups to minimize selection bias. Investigators were not blinded to group allocation. No animals or datasets were excluded from the analyses. Except for RNA sequencing, all experiments were independently repeated at least three times. Sample sizes are provided in the corresponding figure legends.

### 5.2 Animals

C57BL/6J mice were purchased from Huafukang Bioscience (Beijing, China). Female mice (8 weeks old) were used to establish the pregestational diabetes mellitus (PGDM) model. Diabetes was induced by intraperitoneal injection of streptozotocin (STZ; Sigma, #S0130) dissolved in 0.01 M citrate buffer (pH 4.5). STZ selectively destroys pancreatic β‐cells through GLUT2‐mediated uptake and DNA alkylation, resulting in stable hyperglycemia [52]. Depending on the dosing regimen, STZ can be used to generate experimental models of type 1 diabetes mellitus, type 2 diabetes mellitus, and gestational diabetes mellitus [53, 54].

In the present study, mice received STZ (75 mg kg^−1^) once daily for three consecutive days (Figure 1a), producing partial β‐cell depletion and a PGDM model suitable for investigating the effects of maternal hyperglycemia on fetal brain development. Blood glucose levels were measured seven days after the final STZ injection using an Accu‐Chek Aviva Blood Glucose System (Roche, USA). Mice with fasting blood glucose concentrations exceeding 16 mmol L^−1^ (288 mg dL^−1^) were considered diabetic [15, 55]. Age‐matched control mice received vehicle treatment and maintained normoglycemia (4–8 mmol L^−1^) before and throughout pregnancy.

For breeding, two diabetic (or control) females were housed overnight with one wild‐type male. The presence of a vaginal plug the following morning was designated embryonic day 0.5 (E0.5). Maternal blood glucose levels were monitored every six days throughout pregnancy. Pregnant mice were euthanized at E12.5, E15.5, or E18.5 following deep anesthesia with pentobarbital sodium (150 mg kg^−1^, intraperitoneal), and embryos were collected by cesarean section for subsequent analyses.

All animal procedures were approved by the Institutional Animal Care and Use Committee (IACUC) of Jinan University (Approval No. 20210302‐55) and were performed in accordance with institutional guidelines for the care and use of laboratory animals.

### 5.3 Histology and Immunohistochemistry

Embryonic brains were fixed in 4% paraformaldehyde (PFA) at 4°C overnight. For paraffin sections, tissues were dehydrated, embedded in paraffin, and coronally sectioned at 5 µm. Paraffin sections were de‐waxed in xylene and rehydrated. For Nissl staining, sections were stained with 0.5% crystal violet and then dehydrated through graded alcohols. For histological analyses, sections were heated in a microwave oven for antigen retrieval in citrate buffer (10 mM sodium citrate, pH 6.0) before exposure to the primary antibody.

For immunofluorescence on cryosections, fixed brains were cryoprotected sequentially in 15% sucrose for 15 min and 30% sucrose overnight at 4°C. Samples were embedded in Tissue‐Tek Optimal Cutting Temperature (OCT) compound (Sakura, #4583) and coronally sectioned at 20 µm using a cryostat (Leica CM1950).

Cryosections were blocked with 5% heat‐inactivated goat serum in PBS for 1 h at room temperature and incubated with primary antibodies overnight at 4°C. After washing with PBS, sections were incubated with Alexa Fluor 488‐ or 555‐conjugated secondary antibodies (1:1000; Abcam, #ab150078, #ab150077, #ab150113, #ab150114) for 2 h at room temperature in the dark. Nuclei were counterstained with DAPI (1:1000; Beyotime, #C1005) for 30 min, and sections were mounted with Fluoromount‐G mounting medium (SouthernBiotech).

Primary antibodies used in this study were: Sox2 (1:200, Abcam, #ab171380, RRID:AB_2732072; Abcam, #ab97959, RRID:AB_2341193), Tbr2 (1:200, Abcam, #ab23345, RRID:AB_778267), Ctip2 (1:200, Abcam, #ab18465, RRID:AB_2064130), Cux1 (1:200, Abcam, #ab54583, RRID:AB_941209), Foxp1 (1:400, Abcam, #ab16645, RRID:AB_732428), GFP (1:1000, Abcam, #ab13970, RRID:AB_300798), GFP (1:1000, Novus, #NB600‐308, RRID:AB_10003058), Ki67 (1:50, BD Biosciences, #550609, RRID:AB_393778), phospho‐histone H3 (1:400, Cell Signaling Technology, #9713, RRID:AB_823532), γ‐tubulin (1:200, Sigma‐Aldrich, #T6557, RRID:AB_477584), Hes1 (1:1000, HUABIO, #ET1610‐97, RRID:AB_3069983), Tbr1 (1:500, HUABIO, #ET1702‐97, RRID:AB_3070356), Pax6 (1:800, HUABIO, #ET1612‐58, RRID:AB_3070132) and Notch1 (1:500, Bioss, #bsm‐60871R, RRID:AB_3750587).

### 5.4 Edu Incorporation

To label proliferating cells, pregnant dams received an intraperitoneal injection of 5‐ethynyl‐2′‐deoxyuridine (EdU; 100 mg kg^−1^; Beyotime, #C0075S) dissolved in sterile 0.9% saline at E11.5 or E14.5. Embryos were collected 24 h later, and brains were fixed overnight in 4% PFA for subsequent immunofluorescence analyses.

### 5.5 Imaging and Quantification

Images were acquired using an Olympus BX53 fluorescence microscope equipped with cellSens Standard software (version 1.9). Image analysis and quantification were performed using Fiji (ImageJ, version 2.5.0). Positive cells were manually counted from comparable cortical regions using identical imaging parameters for all experimental groups. Quantitative analyses were performed on sections from at least three independent biological replicates, and the corresponding sample sizes are provided in the figure legends.

### 5.6 Western Blot

Total protein was extracted from cultured cells or embryonic mouse cerebral cortices using radioimmunoprecipitation assay (RIPA) buffer (Beyotime, #P0013) supplemented with protease inhibitors. Protein concentrations were determined using the bicinchoninic acid (BCA) protein assay. Equal amounts of protein were separated by 10% SDS‐PAGE and transferred onto polyvinylidene fluoride (PVDF) membranes (Millipore, #03010040001). Membranes were blocked with 5% non‐fat milk in TBST for 1 h at room temperature and incubated with primary antibodies overnight at 4°C. After washing, membranes were incubated with horseradish peroxidase‐conjugated secondary antibodies for 1 h at room temperature. Immunoreactive bands were visualized using BeyoECL Moon chemiluminescent substrate (Beyotime, #P0018) and quantified with Fiji (ImageJ, version 2.5.0).

Primary antibodies used were: Foxp1 (1:1000, Abcam, #ab16645, RRID: AB_732428), Dll1 (1:1000, Cell Signaling Technology, #2588, RRID: AB_2292961), NICD (1:1000, Cell Signaling Technology, #4147, RRID: AB_2153348) and β‐actin (1:5000, Sigma‐Aldrich, #A5441, RRID: AB_476744). Secondary antibodies used were as follows: anti‐mouse and anti‐rabbit IgG (H+L) (1:3000, Proteintech, #SA00001‐1, #SA00001‐2).

### 5.7 RNA Isolation and Quantitative PCR

Total RNA was extracted from embryonic cerebral cortex tissues using TRIzol Reagent (Invitrogen, #15596026CN) according to the manufacturer's instructions. First‐strand cDNA was synthesized using the PrimeScript RT Reagent Kit with gDNA Eraser (Takara, #RR047A). Quantitative PCR (qPCR) was performed using SYBR Green chemistry on a C1000 Touch Thermal Cycler (Bio‐Rad). PCR amplification was carried out under the following conditions: an initial denaturation at 95°C for 2 min, followed by 40 cycles of 95°C for 15 s and 60°C for 30 s. Relative gene expression was normalized to β‐actin and calculated using the 2−ΔΔCq method. Each experiment was performed with three independent biological replicates. Primer sequences are listed in Table S11.

### 5.8 DNA Constructs

Short hairpin RNAs (shRNAs) targeting mouse Foxp1 or Dll1 were cloned into the pLVshRNA‐U6‐PGK‐EGFP vector. The target sequences are listed in Table S12.

For Foxp1 knockdown experiments, equal amounts of shFoxp1‐1 and shFoxp1‐3 plasmids (1.0 mg mL^−1^ total DNA) were pooled and used for in vitro and in vivo experiments. A non‐targeting scrambled shRNA served as the negative control.

For Foxp1 overexpression, the full‐length mouse Foxp1 coding sequence was cloned into the EcoRI/XhoI sites of the pCIG‐IRES‐EGFP vector to generate pCIG‐Foxp1. The radial glial reporter plasmid pBLBP‐EGFP was obtained from Addgene (#63174). All plasmids were purified using the MidiBEST Endotoxin‐Free Plasmid Purification Kit (Takara, #9783).

### 5.9 Cell Culture and Transfection

NIH3T3 cells (ATCC, CRL‐1658) were cultured in Dulbecco's Modified Eagle Medium (DMEM; Gibco, #C11995500BT) supplemented with 10% fetal bovine serum (FBS; Gibco, #C0232) and 1% penicillin‐streptomycin. Cells were maintained at 37°C in a humidified incubator containing 5% CO_2_. Cells were seeded onto 6‐cm culture dishes and transfected at approximately 70% confluence using Lipofectamine 3000 (Invitrogen, #L3000015) according to the manufacturer's protocol. Cells were transfected with pLV‐shControl, pLV‐shFoxp1, pCIG‐EGFP, or pCIG‐Foxp1 plasmids. The culture medium was replaced 4–6 h after transfection, and cells were harvested 48 h later for protein extraction and subsequent analyses.

Cell identity was authenticated by the supplier (ATCC). The cells were not re‐authenticated or routinely tested for mycoplasma contamination after receipt.

### 5.10 In Utero Electroporation

In utero electroporation (IUE) was performed as previously described [56, 57]. Briefly, plasmid DNA (1.0 mg mL^−1^) mixed with 0.05% Fast Green was injected into the lateral ventricle of the left telencephalic vesicle of E14.5 embryos using a glass micropipette. Five square electric pulses (40 V, 50 ms duration, 500 ms intervals) were delivered using a BTX Harvard Apparatus electroporator. Embryonic brains were collected at E15.5, E16.5 or E17.5, fixed overnight in 4% PFA, cryosectioned, and processed for immunofluorescence analysis.

### 5.11 Sholl Analyses

EGFP‐labeled cortical neurons were imaged using an Olympus BX61WI confocal microscope. Dendritic complexity was quantified by Sholl analysis using concentric circles centered on the neuronal soma with 5 µm radial increments. The number of dendritic intersections at each radius was quantified using Fiji (ImageJ). Total dendritic length was measured using the Simple Neurite Tracer plugin in Fiji (National Institutes of Health, USA).

### 5.12 RNA Sequencing and Functional Enrichment Analysis

Total RNA was isolated from embryonic cerebral cortices using TRIzol Reagent (Invitrogen, #15596026CN) according to the manufacturer's instructions. Residual genomic DNA was removed using DNase I (Takara, #RR047A). RNA quality was assessed using an Agilent 2100 Bioanalyzer, and RNA concentration was determined using a NanoDrop ND‐2000 spectrophotometer (Thermo Fisher Scientific). High‐quality RNA samples were submitted to Shanghai Majorbio Bio‐Pharm Technology Co., Ltd. (Shanghai, China) for library preparation and RNA sequencing.

Transcript abundance was quantified as transcripts per million (TPM) using RSEM [58]. Differential gene expression analysis was performed using DESeq2 [59]. Genes with an adjusted *p* value (false discovery rate, FDR) < 0.05 and an absolute log2 fold change (|log2FC|) > 1 were considered differentially expressed.

Functional enrichment analyses, including Gene Ontology (GO), Kyoto Encyclopedia of Genes and Genomes (KEGG), and Disease Ontology enrichment, were performed using the ToppGene Suite [60]. Bonferroni‐adjusted *p* values < 0.05 were considered statistically significant.

Gene Set Enrichment Analysis (GSEA) was performed using the entire ranked transcriptome. Statistical significance was determined using 1000 phenotype permutations, with an FDR *q* value < 0.25 and nominal *p* < 0.05 considered significant.

### 5.13 Bioinformatic Analysis

Putative Foxp1‐binding motifs within the promoter regions of candidate target genes were predicted using the JASPAR database (http://jaspar.genereg.net/). Genes associated with cortical development were retrieved from the GWAS Catalog (https://www.ebi.ac.uk/gwas/). Developmental expression patterns were examined using the Allen Developing Mouse Brain Atlas (https://developingmouse.brain‐map.org/).

### 5.14 Dual Luciferase Reporter Assay

To examine whether Foxp1 regulates Dll1 transcription, a mouse Dll1 promoter fragment (−2000 bp to +200 bp relative to the transcription start site) was cloned upstream of the firefly luciferase reporter in the pGL3 vector. HEK293 cells were seeded into 48‐well plates at a density of 5 × 10^4^ cells per well. At approximately 70% confluence, cells were co‐transfected with 1 µg of either pcDNA3.1 or pcDNA3.1‐Foxp1 expression plasmid, 1 µg of the Dll1 promoter luciferase reporter, and 10 ng of the Renilla luciferase control plasmid using Lipofectamine 3000 (Thermo Fisher Scientific, #L3000015). Transfections were performed in Opti‐MEM (Gibco, #31985070) for 8 h, after which the medium was replaced with complete DMEM. 48 h after transfection, cells were lysed and luciferase activity was measured using the Dual‐Luciferase Reporter Assay System (Promega, #E1910) according to the manufacturer's instructions. Firefly luciferase activity was normalized to Renilla luciferase activity to control for transfection efficiency. Luminescence was measured using a Cytation 5 microplate reader (BioTek). Four independent biological replicates were performed across three experimental batches.

### 5.15 Cleavage under Targets and Tagmentation (CUT & Tag)

CUT&Tag libraries were prepared using the NovoNGS CUT&Tag 4.0 High‐Sensitivity Kit for Illumina (Novoprotein, #N259‐YH01‐01A) according to the manufacturer's instructions. Briefly, dissociated cortical cells isolated from E15.5 embryos (1 × 10^5^ cells per reaction) were incubated with prewashed concanavalin A (ConA)‐coated magnetic beads for 10 min at room temperature. Bead‐bound cells were then incubated with a Foxp1 antibody or rabbit IgG control overnight at 4°C, followed by incubation with the secondary antibody for 1 h at room temperature. After washing, samples were incubated with ChiTag pAG‐Transposome for 1 h at room temperature. Tagmentation was initiated by the addition of MgCl_2_‐containing tagmentation buffer and carried out at 37°C for 1 h. The reaction was terminated using Stop Buffer and Proteinase K, followed by incubation at 55°C for 10 min. DNA fragments were purified using NovoNGS Tagment DNA Extraction Beads, and sequencing libraries were generated by PCR amplification using indexed primers.

CUT&Tag libraries were sequenced on an Illumina NovaSeq platform (paired‐end 150 bp) by Guangzhou Newblue Gene Technology Co., Ltd. (Guangzhou, China). Primary antibodies used for CUT&Tag were Foxp1 (1:50; Cell Signaling Technology, #4402T, RRID: AB_10545755) and rabbit IgG (1:100; Novoprotein, #N269‐01A).

### 5.16 Quantification of Immunofluorescence and Electroporation Data

For immunofluorescence analyses, 300‐µm‐wide cortical columns were selected from comparable anatomical regions for quantification of positive cell numbers and fluorescence intensity. For in utero electroporation experiments, 200‐µm‐wide cortical columns centered within the electroporated region were analyzed. Positive cells were manually counted using Fiji (ImageJ).

For neuronal migration analyses, the cortex was divided into three anatomical regions corresponding to the ventricular zone (VZ), subventricular zone (SVZ), and cortical plate (CP), defined by Sox2, Tbr2, and DAPI staining, respectively. The distribution of EGFP‐positive cells within each region was quantified and expressed as the percentage of total EGFP‐positive cells.

Radial glial cell (RGC) endfoot morphology was evaluated using 20‐µm confocal z‐stack images. Endfeet were classified as club‐shaped, defined as a single endfoot terminating a single radial fiber, or branched, defined as multiple endfeet extending from a single radial fiber [24]. Only radial glial cells with basal processes extending to the pial surface were included in the analysis. Quantification was performed using Fiji (ImageJ) as previously described [61, 62].

### 5.17 Accession Numbers

The published Foxp1 ChIP‐seq dataset analyzed in this study is available in the NCBI Gene Expression Omnibus (GEO) under accession number GSE101632. The RNA‐seq and CUT&Tag sequencing datasets generated during this study have been deposited in the NCBI Sequence Read Archive (SRA) under accession number PRJNA1252857.

### 5.18 Statistical Analysis

Statistical analyses were performed using SPSS (version 13.0). Data are presented as mean ± standard deviation (SD) unless otherwise indicated. Comparisons between two groups were performed using an unpaired, two‐tailed Student's t‐test when data satisfied assumptions of normality and equal variance; otherwise, the Mann‐Whitney U test was used. Comparisons among multiple groups were analyzed using one‐way analysis of variance (ANOVA) followed by the Student–Newman–Keuls post hoc test. A two‐sided *p* value < 0.05 was considered statistically significant. Detailed statistical information, including sample sizes, statistical tests, and exact *p* values, is provided in Tables S1–S10.

## Author Contributions

Conceptualization: **S. Han**, **X. Wang**, **G. Liu**, and **Y. Jin**; Methodology: **L. He** and **Y. Jin**; Investigation: **L. He**, **X. Wang**, **M. He**, and **Y. Shen**; Validation: **X. Zhu** and **H. Liu**; Formal analysis: **L. He** and **W. Xu**; Supervision: **X. Wang**, **S. Han**, and **Y. Jin**; Funding acquisition: **W. Li**, **X. Wang**, **S. Han**, **Y. Jin**, and **L. He**; Visualization: **L. He** and **Y. Jin**; Project administration: **X. Yang** and **B. Li**; Writing – original draft: **L. He** and **X. Wang**; Writing – review & editing: **X. Wang**, **L. He**, **Y. Jin**, and **S. Han**. All authors contributed to analyzing the data and commenting on the manuscript.

## Funding

National Natural Science Foundation of China (81801492, 82271196, and 82301932), Guangdong Basic and Applied Basic Research Foundation (2021A1515012428), Science and Technology Projects in Guangzhou (2025A03J3464, 2024A04J4496, 2024A03J1239, 202102020035, 202201020041), Medical Scientific Research Foundation of Guangdong Province (A2022059), Li Wei's Dongguan Eminent Physician Studio, Dongguan Key Laboratory of Prevention and Treatment of Critical Illness (SYS2024010).

## Ethics Statement

All animal experiments were approved by the Institutional Animal Care and Use Committee (IACUC) of Jinan University (Approval No. 20210302–55) and conducted in accordance with the guidelines for the care and use of laboratory animals.

## Conflicts of Interest

The authors declare no conflicts of interest.

## Supporting information




**Supporting File**: advs78305‐sup‐0001‐SuppMat.docx.

## Data Availability

The data that support the findings of this study are available from the corresponding author upon reasonable request.
