# Supplementary material for: Maternal Hyperglycemia Disrupts Cerebral Cortex Development Through the Foxp1‐Dll1‐Notch Axis
Source: Adv Sci (Weinh). 2026 Oct 26:e78305. Online ahead of print. doi: 10.1002/advs.78305 (PMC13710705; doi:10.1002/advs.78305)
Supplement: Supplementary file 1 [file ADVS-9999-e78305-s001.docx]

Supporting Information

**Maternal Hyperglycemia Disrupts Cerebral Cortex Development through the Foxp1-Dll1-Notch Axis**

Longkai He^1,3, †^, Ya Jin^1, †^, Guosheng Liu^1, †^, Hao Liu^1^, Xinyi Zhu^2^, Weipeng Xu^1^, Wei Li^4^, Meiyao He^2^, Yao Shen^2^, Bingxiao Li^1^, Xuesong Yang^2^, Xiaoyu Wang^2, *^, Shasha Han^1, *^

**† Contributed equally to this work**

***Corresponding Author:** Shasha Han, Ph.D. Xiaoyu Wang, Ph.D.

601 West Huangpu Avenue, Jinan University, Guangzhou, 510632

Email: hanshasha@jnu.edu.cn

Email: wangxiaoyu@jnu.edu.cn

**This PDF file includes:**

Figures S1 to S4

Tables S1 to S12

Figure S1. The proliferation, cell cycle progression, and apoptosis in PGDM cortical NSCs remained unaltered.

**(a)** Box plots showing the number of embryos per pregnant dam in the control and PGDM groups, n = 6 independent dams per group. **(b)** Bar graphs showing the percentages of different types of developmental abnormalities in the control and PGDM groups. **(c)** Quantification of the VZ/SVZ-to-total cortical thickness ratio based on Nissl staining in control and PGDM groups, n = 3 independent dams per group (2 embryos per litter). **(d)** The Sox2⁺ progenitor layer relative to total cortical thickness in control and PGDM groups, n = 3 independent dams per group (2 embryos per litter). **(e)** Quantification of the Sox2⁺ progenitor layer-to-total cortical thickness ratio in control and PGDM groups, n = 3 independent dams per group (2 embryos per litter). (**f**) Representative images of Sox2 and Tbr1 immunostaining in coronal cortical sections at E15.5. (**g, h**) Quantification of Sox2⁺ cells and Tbr1⁺ cells (g), and the Tbr1⁺/Sox2⁺ ratio (h), n = 3 independent dams per group (2 embryos per litter). (**i**) Representative images of Ctip2 and Pax6 double immunostaining in E15.5 embryonic cortex. (**j, k**) Quantification of Pax6⁺ cells and Ctip2⁺ cells (j), and the Ctip2⁺/Pax6⁺ ratio (k), n = 3 independent dams per group (2 embryos per litter). **(l)** Apoptosis assessment via Cleaved Caspase-3 staining (red) at E12.5, E15.5, E18.5 and P1. **(m)** Quantification of Cleaved Caspase-3^+^ cells, n = 6 independent dams per group (2 embryos per litter). **(n)** Co-staining of Sox2 (green) and phosphorylated Histone H3 (pH3, red) in E12.5 and E15.5 cortices. **(o)** Co-labeling of Tbr2 (green) and Ki67 (red). **(p)** Representative images of EdU (red; S-phase marker) and Ki67 (green; proliferative marker) co-staining for cell-cycle analysis. **(q)** Quantification of pH3^+^Sox2⁺ cells among Sox2⁺ population, n = 5 independent dams per group (2 embryos per litter). **(r)** Percentage of Ki67^+^Tbr2⁺ cells among Tbr2⁺ population, n = 5 independent dams per group (2 embryos per litter). **(s)** Quantification of the ratios of EdU⁺Ki67⁻ (postmitotic) and EdU⁺Ki67⁺ (cycling) cells in control and PGDM groups, n = 3 independent dams per group (2 embryos per litter). Detailed sample sizes and statistical analyses are provided in **Tables S2 and S4**. Data are presented as mean ± SD; ****p<*0.001, ***p<*0.01, **p<*0.05, ns = not significant. Scale bars: 25 μm (f), (i), (l) (E12.5 and E15.5), (n), (o) and (p), 50 μm in (l) (E18.5 and P1). Abbreviations: E, embryonic day; P, postnatal day; PGDM, pregestational diabetes mellitus.

**Figure S2. Foxp1 protein and mRNA expression.**

(**a, b**) Foxp1 expression patterns in the control cerebral cortex across different developmental stages (a) and quantitative analysis (b). **(c)** qPCR analysis of *Foxp1* mRNA levels in control and PGDM cortices at E12.5 and E15.5, n = 3 independent dams per group (1 embryo per litter). (d–f) Western blot and qPCR analysis of Foxp1 protein (d, e) and mRNA levels (f) in control cortices at E12.5, E15.5, E18.5 and P1, n = 3 independent dams per group (1 embryo per litter). (g, h) Western blot and qPCR analysis of Foxp1 protein (g) and mRNA levels (h) following shFoxp1 knockdown, n = 3 independent experiments. Detailed sample sizes and statistical analyses are provided in **Table S5**. Data are presented as mean ± SD; ****p<*0.001, ***p<*0.01, **p<*0.05, ns = not significant. Scale bars: 50 μm in (a). Abbreviations: E, embryonic day; P, postnatal day; PGDM, pregestational diabetes mellitus.

**Figure S3. Notch pathway-related gene expression was significantly increased in the PGDM group.**

**(a)** Expression of *Dll1* and *Notch1* mRNAs in the cerebral cortex of control and PGDM groups at E12.5, n = 3 independent dams per group (1 embryo per litter). **(b)** qPCR analysis of *Dll1* and *Notch1* mRNA levels following Foxp1 knockdown or overexpression, n = 3 independent experiments. (**c, d**) Representative immunofluorescence images of Notch1 (c) and Hes1 (d) expression in cortical sections from control and PGDM groups at E15.5 and E18.5. **(e, f)** Quantification of Notch1 (e) and Hes1 (f) fluorescence intensity, n = 3 independent dams per group (2 embryos per litter; 4-5 cells per embryo). (g–i) Western blot (g, h) and qPCR (i) analysis of Dll1 expression after shDll1 knockdown. n = 3 independent experiments. **(j)** Quantification of Dll1 immunoreactivity in cortical sections after Foxp1 knockdown in control and overexpression in PGDM groups, n = 3 independent dams per group (2 embryos per litter; 4-5 cells per embryo). Detailed sample sizes and statistical analyses are provided in **Tables S5 and S9**. Data are presented as mean ± SD; ****p<*0.001, ***p<*0.01, **p<*0.05, ns = not significant. Scale bars: 25 μm in (c) and (d). Abbreviations: E, embryonic day; PGDM, pregestational diabetes mellitus.

**Figure S4. Full-length western blot images for Fig. 6b and Fig. 7k.**
Membranes were probed with anti-Foxp1(a, b), anti-Dll1(b), anti-NICD(c), and anti-β-actin antibodies.

**Table S1. Comparison of blood glucose levels during pregnancy between the control and PGDM groups.**

| **Properties** | | **Control(n)** | **PGDM(n)** | **Statistical test** | **t(df)=value/U value** | **p-value** |
| --- | --- | --- | --- | --- | --- | --- |
| **Fasting glucose (mmol/L)** | | | | |  |  |
|  | Baseline | 5.31 ± 1.17(10) | 5.70 ± 0.79(10) | Student’s t-test | t (18) =0.86 | 0.397 |
|  | Week 1 | 6.25 ± 0.98(10) | 20.72 ± 3.50(10) | Mann–Whitney U test | U = 0 | <0.001*** |
|  | Week 2 | 5.55 ± 1.02 (10) | 20.66 ± 3.51(10) | Mann–Whitney U test | U = 0 | <0.001*** |
|  | Week 3 | 5.94 ± 1.19(10) | 20.99 ± 4.00(10) | Mann–Whitney U test | U = 0 | <0.001*** |
|  | Week 4 | 4.90 ± 0.96(10) | 24.75 ± 2.72(10) | Mann–Whitney U test | U = 0 | <0.001*** |

Data are presented as mean ± standard deviation; df, Degrees of freedom; PGDM, Pregestational diabetes mellitus; SD, Standard deviation; ****p<*0.001, ***p<*0.01 and **p<*0.05. Comparisons between two groups were performed using an unpaired, two-tailed Student’s t-test for data with equal variances or the Mann–Whitney U test for data with unequal variances.

**Table S2. Comparison of embryonic development between the control and PGDM groups.**

| **Properties** | | **Control(n)** | **PGDM(n)** | **Statistical test** | **t(df)=value** | **p-value** |
| --- | --- | --- | --- | --- | --- | --- |
| **Fetal body weight (g)** | | | | |  |  |
|  | E12.5 | 0.08 ± 0.01(6) | 0.05 ± 0.01(6) | Student’s t-test | t (10) =7.32 | <0.001*** |
|  | E15.5 | 0.35 ± 0.01(6) | 0.22 ± 0.03(6) | Student’s t-test | t (10) =9.90 | <0.001*** |
|  | E18.5 | 1.10 ± 0.06(6) | 0.79 ± 0.03(6) | Student’s t-test | t (10) =10.54 | <0.001*** |
|  | P1 | 1.45 ± 0.03(6) | 1.25 ± 0.07(6) | Student’s t-test | t (10) =5.66 | <0.001*** |
| **Fetal brain weight (g)** | | | | |  |  |
|  | E18.5 | 0.29 ± 0.01(6) | 0.23 ± 0.01(6) | Student’s t-test | t (10) =7.19 | <0.001*** |
|  | P1 | 0.39 ± 0.01(6) | 0.33 ± 0.02(6) | Student’s t-test | t (10) =4.70 | 0.001** |
| **Number of embryos** | | | | |  |  |
|  | E12.5 | 6.50 ± 1.05(6) | 5.00 ± 0.89(6) | Student’s t-test | t (10) =2.67 | 0.024* |
|  | E15.5 | 6.33 ± 0.52(6) | 4.67 ± 1.03(6) | Student’s t-test | t (10) =3.54 | 0.005** |
|  | E18.5 | 6.67 ± 1.37(6) | 4.83 ± 0.98(6) | Student’s t-test | t (10) =2.67 | 0.026* |
|  | P1 | 6.00 ± 1.26(6) | 4.50 ± 0.84(6) | Student’s t-test | t (10) =2.42 | 0.036* |

Data are presented as mean ± standard deviation; n=3 dams/group (2 embryos/litter); df, Degrees of freedom; PGDM, Pregestational diabetes mellitus; SD, Standard deviation; ****p<*0.001, ***p<*0.01 and **p<*0.05. Comparisons between two groups were performed using an unpaired, two-tailed Student’s t-test for data with equal variances or the Mann–Whitney U test for data with unequal variances.

**Table S3. Comparison of cortical thickness between the control and PGDM groups.**

| **Properties** | | **Control(n)** | **PGDM(n)** | **Statistical test** | **t(df)=value/U value** | **p-value** |
| --- | --- | --- | --- | --- | --- | --- |
| **The overall thickness of the cerebral cortex (μm)** | | | | |  |  |
|  | E12.5 | 107.20 ± 3.29(6) | 86.95 ± 4.59(6) | Student’s t-test | t (10) =8.77 | <0.001*** |
|  | E15.5 | 305.77 ± 5.44(6) | 200.53 ± 7.59(6) | Student’s t-test | t (10) =27.59 | <0.001*** |
|  | E18.5 | 389.25 ± 6.01(6) | 359.02 ± 18.42(6) | Mann–Whitney U test | U = 1 | 0.008** |
|  | P1 | 406.45 ± 15.60(6) | 375.81 ± 9.81(6) | Student’s t-test | t (10) =4.07 | 0.002** |
| **The thickness of the VZ/SVZ, IZ and CP of the cerebral cortex (μm)** | | | | |  |  |
|  | E15.5 CP | 75.54 ± 4.71(6) | 43.50 ± 3.13(6) | Student’s t-test | t (10) =13.85 | <0.001*** |
|  | E15.5 IZ | 137.86 ± 7.46(6) | 87.63 ± 6.27(6) | Student’s t-test | t (10) =12.62 | <0.001*** |
|  | E15.5 VZ/SVZ | 85.60 ± 5.52(6) | 64.84 ± 2.57(6) | Student’s t-test | t (10) =8.34 | <0.001*** |
|  | E18.5 CP | 209.36 ± 3.33(6) | 178.42 ± 6.71(6) | Student’s t-test | t (10) =10.1 | <0.001*** |
|  | E18.5 IZ | 123.75 ± 9.40(6) | 111.25 ± 17.11(6) | Student’s t-test | t (10) =1.56 | 0.147 |
|  | E18.5 VZ/SVZ | 41.19 ± 6.72(6) | 61.36 ± 4.11(6) | Student’s t-test | t (10) =6.26 | <0.001*** |
|  | P1 CP | 280.01 ± 30.86(6) | 231.71 ± 15.80(6) | Student’s t-test | t (10) =3.41 | 0.006** |
|  | P1 IZ | 98.59 ± 25.58(6) | 100.11 ± 10.96(6) | Mann–Whitney U test | U = 17 | 0.896 |
|  | P1 VZ/SVZ | 22.16 ± 4.00(6) | 39.45 ± 7.54(6) | Student’s t-test | t (10) =4.96 | 0.001** |
| **Percentage of the proportionate thickness in overall cerebral cortex (%)** | | | | |  |  |
|  | E15.5 CP | 24.71 ± 1.61(6) | 21.07 ± 1.17(6) | Student’s t-test | t (10) =4.47 | 0.001** |
|  | E15.5 IZ | 45.09 ± 2.46(6) | 44.05 ± 3.03(6) | Student’s t-test | t (10) =0.65 | 0.529 |
|  | E15.5 VZ/SVZ | 28.01 ± 1.93(6) | 32.70 ± 2.40(6) | Student’s t-test | t (10) =-3.73 | 0.004** |
|  | E18.5 CP | 55.36 ± 3.22(6) | 49.76 ± 2.40(6) | Student’s t-test | t (10) =3.40 | 0.007** |
|  | E18.5 IZ | 32.74 ± 3.40(6) | 30.88 ± 3.79(6) | Student’s t-test | t (10) =0.89 | 0.392 |
|  | E18.5 VZ/SVZ | 10.96 ± 2.40(6) | 17.12 ± 1.43(6) | Student’s t-test | t (10) =5.38 | <0.001*** |
|  | P1 CP | 68.79 ± 5.89(6) | 61.65 ± 3.85(6) | Student’s t-test | t (10) =2.47 | 0.032* |
|  | P1 IZ | 25.48 ± 7.69(6) | 26.62 ± 2.68(6) | Mann–Whitney U test | U = 15 | 0.743 |
|  | P1 VZ/SVZ | 5.44 ± 0.92(6) | 10.51 ± 2.12(6) | Student’s t-test | t (10) =5.36 | 0.001** |

Data are presented as mean ± standard deviation; n=3 dams/group (2 embryos/litter); CP, Cortical plate; df, Degrees of freedom; IZ, Intermediate zone; PGDM, Pregestational diabetes mellitus; SVZ, Subventricular zone; VZ, Ventricular zone; SD, Standard deviation; ****p<*0.001, ***p<*0.01 and **p<*0.05. Comparisons between two groups were performed using an unpaired, two-tailed Student’s t-test for data with equal variances or the Mann–Whitney U test for data with unequal variances.

**Table S4. Comparison of the differentiation, asymmetric division, migration, and dendritic development between the control and PGDM groups.**

| **Properties** | | **Control(n)** | **PGDM(n)** | **Statistical test** | **t(df)=value/U value** | **p-value** |
| --- | --- | --- | --- | --- | --- | --- |
| **Quantification of Sox2⁺ cells** | | | | | | |
|  | E12.5 | 186.32 ± 15.91(6) | 229.62 ± 16.22(6) | Student’s t-test | t (10) =4.66 | 0.001** |
|  | E15.5 | 99.52 ± 9.56(6) | 130.02 ± 11.51(6) | Student’s t-test | t (10) =4.99 | 0.006** |
|  | E18.5 | 87.43 ± 11.21(6) | 105.09 ± 13.10(6) | Student’s t-test | t (10) =2.50 | 0.031* |
|  | P1 | 50.01 ± 8.23(6) | 62.16 ± 7.12(6) | Student’s t-test | t (10) =2.73 | 0.021* |
| **Quantification of Tbr2⁺ cells** | | | | | | |
|  | E12.5 | 71.92 ± 11.87(6) | 54.14 ± 8.81(6) | Student’s t-test | t (10) =2.94 | 0.011* |
|  | E15.5 | 89.82 ± 10.62(6) | 75.96 ± 7.79(6) | Student’s t-test | t (10) =2.58 | 0.036* |
|  | E18.5 | 43.87 ± 3.74(6) | 36.50 ± 6.38(6) | Student’s t-test | t (10) =2.43 | 0.035* |
|  | P1 | 21.59 ± 3.22(6) | 17.04 ± 2.52(6) | Student’s t-test | t (10) =2.72 | 0.021* |
| **The ratio of Tbr2⁺ to Sox2⁺ cells** | | | | | | |
|  | E12.5 | 0.38 ± 0.05(6) | 0.23 ± 0.04(6) | Student’s t-test | t (10) =5.41 | <0.001*** |
|  | E15.5 | 0.90 ± 0.12(6) | 0.58 ± 0.08(6) | Student’s t-test | t (10) =5.34 | <0.001*** |
|  | E18.5 | 0.50 ± 0.04(6) | 0.34 ± 0.05(6) | Student’s t-test | t (10) =5.31 | <0.001*** |
|  | P1 | 0.43 ± 0.03(6) | 0.27 ± 0.03(6) | Student’s t-test | t (10) =8.53 | <0.001*** |
| **Quantification of Sox2⁺, Tbr1⁺ and the ratio of Tbr1⁺ to Sox2⁺ cells** | | | | | | |
|  | Sox2 | 95.81 ± 6.34(6) | 119.30 ± 5.60(6) | Student’s t-test | t (10) =6.79 | <0.001*** |
|  | Tbr1 | 186.14 ± 11.12(6) | 151.67 ± 6.78(6) | Student’s t-test | t (10) =6.47 | <0.001*** |
|  | Tbr1/Sox2 | 1.94 ± 0.12(6) | 1.27 ± 0.09(6) | Student’s t-test | t (10) =10.05 | <0.001*** |
| **Quantification of Pax6⁺, Ctip2⁺ and the ratio of Ctip2⁺ to Pax6⁺ cells** | | | | | | |
|  | Pax6 | 109.08 ± 4.65(6) | 127.84 ± 2.98(6) | Student’s t-test | t (10) =8.31 | <0.001*** |
|  | Ctip2 | 130.50 ± 6.98(6) | 107.95 ± 7.17(6) | Student’s t-test | t (10) =5.51 | <0.001*** |
|  | Ctip2/Pax6 | 1.19 ± 0.09(6) | 0.84 ± 0.07(6) | Student’s t-test | t (10) =7.35 | <0.001*** |
| **Quantification of Sox2⁺, Ctip2⁺ and the ratio of Ctip2⁺ to Sox2⁺ cells** | | | | | | |
|  | Sox2 | 98.66 ± 12.51(8) | 126.55 ± 21.51(8) | Student’s t-test | t (14) =3.16 | 0.007** |
|  | Ctip2 | 129.64 ± 22.03(8) | 100.26 ± 28.33(8) | Student’s t-test | t (14) =2.31 | 0.036* |
|  | Ctip2/Sox2 | 1.31 ± 0.13(8) | 0.81 ± 0.25(8) | Mann–Whitney U test | U = 0 | 0.001** |
| **Quantification of Ctip2⁺ and Cux1⁺ cells** | | | | |  |  |
|  | E18.5 Ctip2 | 170.66 ± 14.82(8) | 151.67 ± 10.34(8) | Student’s t-test | t (14) =2.97 | 0.009** |
|  | P1 Ctip2 | 154.30 ± 12.40(8) | 133.95 ± 10.54(8) | Student’s t-test | t (14) =3.54 | 0.003** |
|  | E18.5 Cux1 | 166.65 ± 22.59(8) | 105.11 ± 9.91(8) | Student’s t-test | t (14) =7.06 | <0.001*** |
|  | P1 Cux1 | 228.90 ± 16.63(8) | 197.81 ± 19.16(8) | Student’s t-test | t (14) =3.46 | 0.004** |
| **Quantification of symmetrical and asymmetric division** | | | | | | |
|  | Division angles | 58.55 ± 24.41(131) | 61.49 ± 24.98(131) | Student’s t-test | t (260) =0.96 | 0.336 |
|  | Vertical fold changes | 1.00 ± 0.22(17) | 1.16 ± 0.16(17) | Student’s t-test | t (32) =2.49 | 0.018* |
|  | Non-vertical fold changes | 1.00 ± 0.25(17) | 0.80 ± 0.17(17) | Student’s t-test | t (32) =2.69 | 0.011* |
| **Quantification of NSC migration** | | | | | | |
|  | EGFP⁺ in CP | 29.77 ± 3.74(6) | 25.34 ± 2.66(6) | Student’s t-test | t (10) =2.36 | 0.039* |
|  | EGFP⁺ in IZ | 33.46 ± 5.78(6) | 32.66 ± 1.53(6) | Student’s t-test | t (10) =0.33 | 0.748 |
|  | EGFP⁺ in VZ/SVZ | 36.75 ± 2.81(6) | 41.99 ± 3.13(6) | Student’s t-test | t (10) =3.05 | 0.012* |
|  | Leading process length | 71.85 ± 15.30(25) | 40.10 ± 11.59(25) | Student’s t-test | t (48) =8.26 | <0.001*** |
|  | Club like | 62.20 ± 5.98(6) | 63.89 ± 3.31(6) | Student’s t-test | t (10) =0.60 | 0.56 |
|  | Branched | 37.79 ± 5.98(6) | 36.10 ± 3.31(6) | Student’s t-test | t (10) =0.60 | 0.56 |
| **Quantification of dendritic development** | | | | |  |  |
|  | dendritic complexity | | | |  |  |
|  | 20μm | 1.00 ± 0.00(6) | 1.00 ± 0.00(6) | Student’s t-test | t (10) =0.00 | 1 |
|  | 40μm | 1.00 ± 0.00(6) | 1.00 ± 0.00(6) | Student’s t-test | t (10) =0.00 | 1 |
|  | 60μm | 1.50 ± 0.84(6) | 1.33 ± 0.51(6) | Student’s t-test | t (10) =0.42 | 0.687 |
|  | 80μm | 3.00 ± 0.63(6) | 2.00 ± 0.89(6) | Student’s t-test | t (10) =2.24 | 0.049* |
|  | 100μm | 3.67 ± 0.82(6) | 2.17 ± 0.41(6) | Student’s t-test | t (10) =4.03 | 0.002** |
|  | 120μm | 4.00 ± 0.89(6) | 1.17 ± 0.41(6) | Student’s t-test | t (10) =7.06 | <0.001*** |
|  | 140μm | 1.17 ± 1.47(6) | 0.33 ± 0.52(6) | Mann–Whitney U test | U = 13 | 0.485 |
|  | Dendritic length | 461.51 ± 75.06(14) | 333.95 ± 60.01(14) | Student’s t-test | t (26) =4.96 | <0.001*** |
| **Quantification of cell proliferation** | | | | |  |  |
|  | E12.5(pH3) | 3.91 ± 0.67(10) | 3.51 ± 0.61(10) | Student’s t-test | t (18) =1.36 | 0.189 |
|  | E15.5(pH3) | 3.66 ± 0.70(10) | 3.67 ± 0.95(10) | Student’s t-test | t (18) =0.02 | 0.98 |
|  | E12.5(Ki67) | 47.51 ± 5.16(10) | 48.38 ± 5.70(10) | Student’s t-test | t (18) =0.35 | 0.725 |
|  | E15.5(Ki67) | 40.18 ± 4.65(10) | 40.03 ± 4.53(10) | Student’s t-test | t (18) =0.07 | 0.944 |
| **Quantification of cell cycle** | | | | |  |  |
|  | E15.5 | 73.18 ± 6.40(6) | 72.40 ± 9.07(6) | Student’s t-test | t (10) =0.17 | 0.867 |
| **Quantification of cell apoptosis** | | | | |  |  |
|  | E12.5 | 0.83 ± 0.83(12) | 0.75 ± 0.75(12) | Student’s t-test | t (22) =0.26 | 0.800 |
|  | E15.5 | 0.67 ± 0.78(12) | 0.58 ± 0.79(12) | Student’s t-test | t (22) =0.26 | 0.797 |
|  | E18.5 | 2.00 ± 1.04(12) | 1.92 ± 1.24(12) | Student’s t-test | t (22) =0.18 | 0.860 |
|  | P1 | 5.92 ± 1.73(12) | 6.17 ± 1.53(12) | Student’s t-test | t (22) =0.38 | 0.711 |

Data are presented as mean ± standard deviation; n=3-6 dams/group (2 embryos/litter; 4-5 cells/embryo); CP, Cortical plate; df, Degrees of freedom; IZ, Intermediate zone; PGDM, Pregestational diabetes mellitus; SVZ, Subventricular zone; VZ, Ventricular zone; SD, Standard deviation; ****p<*0.001, ***p<*0.01 and **p<*0.05. Comparisons between two groups were performed using an unpaired, two-tailed Student’s t-test for data with equal variances or the Mann–Whitney U test for data with unequal variances.

**Table S5. Protein and RNA expression levels between the control and PGDM groups.**

| **Properties** | | **Control(n)** | **PGDM(n)** | **Statistical test** | **t(df)=value/U value** | **p-value** |
| --- | --- | --- | --- | --- | --- | --- |
| **Expression pattern of mRNA** | | | | |  |  |
|  | Neurog2 | 1.00 ± 0.24(9) | 1.49 ± 0.14(9) | Mann–Whitney U test | U = 3 | 0.001** |
|  | Nr2f1 | 1.00 ± 0.19(9) | 1.32 ± 0.18(9) | Student’s t-test | t (16) =3.54 | 0.002** |
|  | Tubb3 | 1.00 ± 0.11(9) | 0.82 ± 0.08(9) | Student’s t-test | t (16) =3.59 | 0.002** |
|  | Reln | 1.00 ± 0.17(9) | 0.81 ± 0.10(9) | Student’s t-test | t (16) =2.67 | 0.016* |
|  | Foxp1 | 1.00 ± 0.14(9) | 0.44 ± 0.13(9) | Student’s t-test | t (16) =8.41 | <0.001*** |
|  | Dab1 | 1.00 ± 0.15(9) | 0.85 ± 0.08(9) | Mann–Whitney U test | U = 20 | 0.077 |
|  | Robo1 | 1.00 ± 0.18(9) | 0.86 ± 0.11(9) | Mann–Whitney U test | U = 61 | 0.077 |
|  | Dll1 E12.5 | 1.00 ± 0.08(3) | 1.97 ± 0.10(3) | Student’s t-test | t (4) =12.65 | <0.001*** |
|  | Foxp1 E12.5 | 1.00 ± 0.09(3) | 0.58 ± 0.12(3) | Student’s t-test | t (4) =4.43 | 0.01* |
|  | Foxp1 E15.5 | 1.00 ± 0.03(3) | 0.48 ± 0.06(3) | Student’s t-test | t (4) =12.58 | <0.001*** |
|  | Notch1 E12.5 | 1.00 ± 0.06(3) | 1.58 ± 0.16(3) | Student’s t-test | t (4) =5.67 | 0.005** |
|  | Hes1 | 1.00 ± 0.14(12) | 1.33 ± 0.30(12) | Student’s t-test | t (22) =3.30 | 0.003** |
|  | Hes5 | 1.02 ± 0.24(12) | 1.37 ± 0.26(12) | Student’s t-test | t (22) =3.37 | 0.002** |
|  | Hey1 | 1.00 ± 0.06(12) | 1.58 ± 0.18(12) | Mann–Whitney U test | U = 0 | 0.001** |
|  | Hey2 | 1.02 ± 0.23(12) | 1.10 ± 0.15(12) | Student’s t-test | t (22) =1.04 | 0.31 |
| **Expression pattern of the protein** | | | | |  |  |
|  | DLL1 E12.5 | 1.00 ± 0.01(3) | 1.34 ± 0.11(3) | Student’s t-test | t (4) =5.37 | 0.006** |
|  | NICD E12.5 | 1.00 ± 0.22(3) | 1.70 ± 0.15(3) | Student’s t-test | t (4) =4.48 | 0.006** |
|  | FOXP1 E12.5 | 1.00 ± 0.10(3) | 0.67 ± 0.15(3) | Student’s t-test | t (4) =3.11 | 0.03* |
|  | FOXP1 E15.5 | 1.00 ± 0.11(3) | 0.47 ± 0.06(3) | Student’s t-test | t (4) =7.00 | 0.002** |

Data are presented as mean ± standard deviation; n=3-6 dams/group (1-2 embryos/litter); df, Degrees of freedom; PGDM, Pregestational diabetes mellitus; SD, Standard deviation; ****p<*0.001, ***p<*0.01 and **p<*0.05. Comparisons between two groups were performed using an unpaired, two-tailed Student’s t-test for data with equal variances or the Mann–Whitney U test for data with unequal variances.

**Table S6. Comparison of Foxp1 fluorescence intensity expression levels between the control and PGDM groups.**

| **Properties** | | **Control(n)** | **PGDM(n)** | **Statistical test** | **t(df)=value/U value** | **p-value** |
| --- | --- | --- | --- | --- | --- | --- |
| **Expression pattern of Foxp1 fluorescence intensity** | | | | | | |
|  | E12.5 | 1.00 ± 0.09(6) | 0.43 ± 0.04(6) | Student’s t-test | t (10) =14.48 | <0.001*** |
|  | E15.5 | 1.00 ± 0.13(6) | 0.79 ± 0.07(6) | Student’s t-test | t (10) =3.41 | 0.007** |
|  | E18.5 V-VI | 1.00 ± 0.14(6) | 0.81 ± 0.05(6) | Student’s t-test | t (10) =3.11 | 0.02* |
|  | E18.5 II-IV | 1.00 ± 0.11(6) | 0.69 ± 0.11(6) | Student’s t-test | t (10) =4.93 | 0.001** |
|  | E18.5 I | 1.00 ± 0.05(6) | 0.98 ± 0.05(6) | Student’s t-test | t (10) =0.75 | 0.47 |
|  | P1 V-VI | 1.00 ± 0.12(6) | 0.81 ± 0.11(6) | Student’s t-test | t (10) =2.96 | 0.014* |
|  | P1 II-IV | 1.00 ± 0.13(6) | 0.69 ± 0.05(6) | Student’s t-test | t (10) =5.42 | <0.001*** |
|  | P1 I | 1.00 ± 0.26(6) | 0.76 ± 0.21(6) | Student’s t-test | t (10) =1.68 | 0.123 |
|  | E12.5 apical cells | 1.00 ± 0.08(22) | 0.41 ± 0.05(22) | Mann–Whitney U test | U = 0 | <0.001*** |
|  | Tuj1⁺ cells | 1.00 ± 0.15(60) | 0.61 ± 0.08(60) | Mann–Whitney U test | U = 2 | <0.001*** |

Data are presented as mean ± standard deviation; n=3 dams/group (2 embryos/litter; 3-10 cells/embryo); df, Degrees of freedom; PGDM, Pregestational diabetes mellitus; SD, Standard deviation; ****p<*0.001, ***p<*0.01 and **p<*0.05. Comparisons between two groups were performed using an unpaired, two-tailed Student’s t-test for data with equal variances or the Mann–Whitney U test for data with unequal variances.

**Table S7. Comparison of the differentiation, asymmetric division, migration, and dendritic development in the control and shFoxp1 groups.**

| **Properties** | | **Control(n)** | **shFoxp1(n)** | **Statistical test** | **t(df)=value/U value** | **p-value** |
| --- | --- | --- | --- | --- | --- | --- |
| **Quantification of Sox2⁺ cells** | | | | | | |
|  |  | 43.29±4.39(6) | 52.63±4.18(6) | Student’s t-test | t (10) =3.76 | 0.003** |
| **Quantification of Tbr2⁺ cells** | | | |  |  |  |
|  |  | 18.48±3.44(6) | 13.44±2.86(6) | Student’s t-test | t (10) =2.97 | 0.013* |
| **Quantification of Sox2⁺ EGFP⁺ cells with apical attachment** | | | | | | |
|  |  | 7.83±1.16(6) | 10.50±2.25(6) | Student’s t-test | t (10) =2.56 | 0.027* |
| **Quantification of NSC migration** | | | | | | |
|  | CP | 13.93±3.78(6) | 2.98±1.58(6) | Student’s t-test | t (10) =6.53 | <0.001*** |
|  | IZ | 45.36±2.72(6) | 44.89±3.61(6) | Student’s t-test | t (10) =0.25 | 0.804 |
|  | VZ/SVZ | 40.70±3.53(6) | 52.12±4.74(6) | Student’s t-test | t (10) =4.72 | 0.001** |
| **Quantification of Ctip2⁺ cells** | | | | | | |
|  |  | 6.53±1.72(6) | 2.07±0.97(6) | Student’s t-test | t (10) =5.50 | <0.001*** |

Data are presented as mean ± standard deviation; n=3-6 dams/group (2 embryos/litter); CP, Cortical plate; df, Degrees of freedom; IZ, Intermediate zone; PGDM, Pregestational diabetes mellitus; SVZ, Subventricular zone; VZ, Ventricular zone; SD, Standard deviation; ****p<*0.001, ***p<*0.01 and **p<*0.05. Comparisons between two groups were performed using an unpaired, two-tailed Student’s t-test for data with equal variances or the Mann–Whitney U test for data with unequal variances.

**Table S8. Comparison of the differentiation, asymmetric division, migration, and dendritic development in the control, control+Foxp1, PGDM, and PGDM+Foxp1 groups.**

| **Properties** | | **Control(n)** | **Control+Foxp1(n)** | **PGDM(n)** | **PGDM+Foxp1 (n)** | **Statistical test** | **p-value** |
| --- | --- | --- | --- | --- | --- | --- | --- |
| **Quantification of Sox2⁺ cells** | | | | | | |  |
|  |  | 49.19 ± 6.35(6) | / | 62.90 ± 7.01(6) | / | LSD | <0.001*** |
|  |  | 49.19 ± 6.35(6) | 41.54 ± 1.81(6) | / | / | LSD | 0.030* |
|  |  | 49.19 ± 6.35(6) | / | / | 53.41 ± 5.91(6) | LSD | 0.211 |
|  |  | / | / | 62.90 ± 7.01(6) | 53.41 ± 5.91(6) | LSD | 0.009** |
| **Quantification of Tbr2⁺ cells** | | | | | | |  |
|  |  | 26.76 ± 5.82(6) | / | 19.04 ± 2.74(6) | / | LSD | 0.017* |
|  |  | 26.76 ± 5.82(6) | 33.32 ± 4.38(6) | / | / | LSD | 0.039* |
|  |  | 26.76 ± 5.82(6) | / | / | 26.35 ± 6.71(6) | LSD | 0.890 |
|  |  | / | / | 19.04 ± 2.74(6) | 26.35 ± 6.71(6) | LSD | 0.023* |
| **Quantification of Sox2⁺ EGFP⁺ cells with apical attachment** | | | | | | |  |
|  |  | 7.00 ± 1.78(6) |  | 9.67 ± 1.36(6) | / | LSD | 0.005** |
|  |  | 7.00 ± 1.78(6) | 5.33 ± 1.03(6) | / | / | LSD | 0.061 |
|  |  | 7.00 ± 1.78(6) | / | / | 7.50 ± 1.51(6) | LSD | 0.558 |
|  |  | / | / | 9.67 ± 1.36(6) | 7.50 ± 1.51(6) | LSD | 0.018* |
| **Percentage of symmetric cell division** | | | | | | |  |
|  |  | 65.27 ± 5.64(6) | / | 73.61 ± 6.27(6) | / | LSD | 0.034* |
|  |  | 65.27 ± 5.64(6) | 50.76 ± 6.02(6) | / | / | LSD | 0.001** |
|  |  | / | / | 73.61 ± 6.27(6) | 63.49 ± 6.70(6) | LSD | 0.013* |
|  |  | 65.27 ± 5.64(6) | / | / | 63.49 ± 6.70(6) | LSD | 0.626 |
| **Quantification of NSC migration** | | | | | | |  |
|  | VZ/SVZ | 34.34 ± 3.94(6) |  | 41.09 ± 6.30(6) | / | LSD | 0.018* |
|  |  | 34.34 ± 3.94(6) | 28.31 ± 4.32(6) | / | / | LSD | 0.032* |
|  |  | / | / | 41.09 ± 6.30(6) | 37.53 ± 2.83(6) | LSD | 0.188 |
|  |  | 34.34 ± 3.94(6) | / | / | 37.53 ± 2.83(6) | LSD | 0.237 |
|  | IZ | 33.86 ± 5.35(6) | / | 35.10 ± 3.84(6) | / | LSD | 0.650 |
|  |  | 33.86 ± 5.35(6) | 33.17 ± 5.70(6) | / | / | LSD | 0.801 |
|  |  | / | / | 35.10 ± 3.84(6) | 31.03 ± 3.29(6) | LSD | 0.146 |
|  |  | 33.86 ± 5.35(6) | / | / | 31.03 ± 3.29(6) | LSD | 0.306 |
|  | CP | 31.79 ± 5.97(6) | / | 23.80 ± 5.28(6) | / | LSD | 0.007** |
|  |  | 31.79 ± 5.97(6) | 38.51 ± 3.41(6) | / | / | LSD | 0.020* |
|  |  | / | / | 23.80 ± 5.28(6) | 31.43 ± 3.15(6) | LSD | 0.010* |
|  |  | 31.79 ± 5.97(6) | / | / | 31.43 ± 3.15(6) | LSD | 0.894 |
|  | Leading process length | 64.74 ± 13.81(25) | / | 36.92 ± 12.27(25) | / | LSD | <0.001*** |
|  |  | 64.74 ± 13.81(25) | 63.67 ± 13.67(25) | / | / | LSD | 0.781 |
|  |  | / | / | 36.92 ± 12.27(25) | 51.98 ± 14.22(25) | LSD | <0.001*** |
|  |  | 64.74 ± 13.81(25) | / | / | 51.98 ± 14.22(25) | LSD | 0.001** |
| **Dendritic development** | | | | | |  |  |
|  | Dendritic complexity | | | | | |  |
|  | 20μm | 1.00 ± 0.00(6) | / | 1.00 ± 0.00(6) | / | LSD | 1 |
|  | 20μm | 1.00 ± 0.00(6) | 1.17 ± 0.41(6) | / | / | LSD | 0.173 |
|  | 20μm | / | / | 1.00 ± 0.00(6) | 1.00 ± 0.00(6) | LSD | 1 |
|  | 20μm | 1.00 ± 0.00(6) | / | / | 1.00 ± 0.00(6) | LSD | 1 |
|  | 40μm | 1.33 ± 0.51(6) | / | 1.17 ± 0.41(6) | / | LSD | 0.465 |
|  | 40μm | 1.33 ± 0.51(6) | 1.17 ± 0.41(6) | / | / | LSD | 0.465 |
|  | 40μm | / | / | 1.17 ± 0.41(6) | 1.00 ± 0.00(6) | LSD | 0.465 |
|  | 40μm | 1.33 ± 0.51(6) | / | / | 1.00 ± 0.00(6) | LSD | 0.152 |
|  | 60μm | 2.50 ± 0.55(6) | / | 1.33 ± 0.52(6) | / | LSD | 0.002** |
|  | 60μm | 2.50 ± 0.55(6) | 2.00 ± 0.63(6) | / | / | LSD | 0.135 |
|  | 60μm | / | / | 1.33 ± 0.52(6) | 1.33 ± 0.52(6) | LSD | 1 |
|  | 60μm | 2.50 ± 0.55(6) | / | / | 1.33 ± 0.52(6) | LSD | 0.002** |
|  | 80μm | 3.83 ± 1.47(6) | / | 1.50 ± 0.54(6) | / | LSD | 0.001** |
|  | 80μm | 3.83 ± 1.47(6) | 3.17 ± 1.33(6) | / | / | LSD | 0.296 |
|  | 80μm | / | / | 1.50 ± 0.54(6) | 3.00 ± 0.63(6) | LSD | 0.025* |
|  | 80μm | 3.83 ± 1.47(6) | / | / | 3.00 ± 0.63(6) | LSD | 0.195 |
|  | 100μm | 4.50 ± 0.83(6) | / | 1.83 ± 0.41(6) | / | LSD | <0.001*** |
|  | 100μm | 4.50 ± 0.83(6) | 4.00 ± 1.10(6) | / | / | LSD | 0.342 |
|  | 100μm | / | / | 1.83 ± 0.41(6) | 4.50 ± 1.05(6) | LSD | <0.001*** |
|  | 100μm | 4.50 ± 0.83(6) | / | / | 4.50 ± 1.05(6) | LSD | 1 |
|  | 120μm | 2.50 ± 1.38(6) | / | 1.67 ± 0.82(6) | / | LSD | 0.211 |
|  | 120μm | 2.50 ± 1.38(6) | 3.33 ± 1.03(6) | / | / | LSD | 0.211 |
|  | 120μm | / | / | 1.67 ± 0.82(6) | 3.83 ± 1.17(6) | LSD | 0.003** |
|  | 120μm | 2.50 ± 1.38(6) | / | / | 3.83 ± 1.17(6) | LSD | 0.052 |
|  | 140μm | 0.67 ± 1.21(6) | / | 1.17 ± 0.41(6) | / | LSD | 0.479 |
|  | 140μm | 0.67 ± 1.21(6) | 1.67 ± 1.63(6) | / | / | LSD | 0.165 |
|  | 140μm | / | / | 1.17 ± 0.41(6) | 1.67 ± 1.21(6) | LSD | 0.479 |
|  | 140μm | 0.67 ± 1.21(6) | / | / | 1.67 ± 1.21(6) | LSD | 0.165 |
|  | Dendritic length | 440.25 ± 84.50(14) | / | 331.75 ± 76.91(14) | / | LSD | <0.001*** |
|  |  | 440.25 ± 84.50(14) | 453.49 ± 70.72(14) | / | / | LSD | 0.636 |
|  |  | / | / | 331.75 ± 76.91(14) | 434.467± 60.03(14) | LSD | <0.001*** |
|  |  | 440.25 ± 84.50(14) | / | / | 434.467± 60.03(14) | LSD | 0.836 |

Data are presented as mean ± standard deviation; n=3 dams/group (2 embryos/litter; 2-5 cells/embryo); CP, Cortical plate; df, Degrees of freedom; IZ, Intermediate zone; PGDM, Pregestational diabetes mellitus; SVZ, Subventricular zone; VZ, Ventricular zone; SD, Standard deviation; ****p<*0.001, ***p<*0.01 and **p<*0.05. For comparisons involving multiple groups, one-way analysis of variance (ANOVA) was employed, followed by the Student–Newman–Keuls post hoc test.

**Table S9. Fluorescence intensity expression levels between the control and PGDM groups.**

| **Properties** | | **Control(n)** | **PGDM(n)** | **Statistical test** | **T (df) = value/U value** | **p-value** |
| --- | --- | --- | --- | --- | --- | --- |
| **Expression pattern of Dll1 fluorescence intensity** | | | | |  |  |
|  |  | 1.00 ± 0.20(6) | 2.16 ± 0.41(6) | Student’s t-test | t (10) =6.20 | <0.001*** |
| **Expression pattern of Notch1 fluorescence intensity** | | | | |  |  |
|  | E15.5 | 1.00 ± 0.21(25) | 1.32 ± 0.24(25) | Student’s t-test | t (48) =4.99 | <0.001*** |
|  | E18.5 | 1.00 ± 0.20(25) | 1.15 ± 0.19(25) | Student’s t-test | t (48) =2.76 | 0.008** |
| **Expression pattern of Hes1 fluorescence intensity** | | | | |  |  |
|  | E15.5 | 1.00 ± 0.14(25) | 1.30 ± 0.25(25) | Mann–Whitney U test | U = 101.5 | <0.001*** |
|  | E18.5 | 1.00 ± 0.20(25) | 1.30 ± 0.23(25) | Student’s t-test | t (48) =4.92 | <0.001*** |

Data are presented as mean ± standard deviation; n=3 dams/group (2 embryos/litter; 4-5 cells/embryo); PGDM, Pregestational diabetes mellitus; SD, Standard deviation; ****p<*0.001, ***p<*0.01 and **p<*0.05. Comparisons between two groups were performed using an unpaired, two-tailed Student’s t-test for data with equal variances or the Mann–Whitney U test for data with unequal variances.

**Table S10. Comparison of the differentiation and migration in the control, PGDM, and PGDM+shDll1 groups.**

| **Properties** | | **Control(n)** | **PGDM(n)** | **PGDM+shDll1 (n)** | **Statistical test** | **p-value** |
| --- | --- | --- | --- | --- | --- | --- |
| **Quantification of Sox2⁺ cells** | | | | | |  |
|  |  | 51.64 ± 5.15(6) | 62.92 ± 7.90(6) | / | LSD | 0.006** |
|  |  | 51.64 ± 5.15(6) | / | 58.20 ± 4.94(6) | LSD | 0.084 |
|  |  | / | 62.92 ± 7.90(6) | 58.20 ± 4.94(6) | LSD | 0.204 |
| **Quantification of Tbr2⁺ cells** | | | | | |  |
|  |  | 27.10 ± 4.40(6) | 20.90 ± 4.22(6) | / | LSD | 0.021* |
|  |  | 27.10 ± 4.40(6) | / | 22.50 ± 3.87(6) | LSD | 0.076 |
|  |  | / | 20.90 ± 4.22(6) | 22.50 ± 3.87(6) | LSD | 0.517 |
| **Quantification of NSC migration** | | | | | |  |
|  | VZ/SVZ | 32.36 ± 2.90(6) | 38.07 ± 1.90(6) | / | LSD | 0.003** |
|  |  | / | 38.07 ± 1.90(6) | 34.89 ± 3.35(6) | LSD | 0.067 |
|  |  | 32.36 ± 2.90(6) | / | 34.89 ± 3.35(6) | LSD | 0.137 |
|  | IZ | 40.50 ± 4.47(6) | 39.88 ± 5.02(6) | / | LSD | 0.808 |
|  |  | / | 39.88 ± 5.02(6) | 38.36 ± 3.50(6) | LSD | 0.557 |
|  |  | 40.50 ± 4.47(6) | / | 38.36 ± 3.50(6) | LSD | 0.401 |
|  | CP | 27.12 ± 3.14(6) | 22.04 ± 3.49(6) | / | LSD | 0.007** |
|  |  | / | 22.04 ± 3.49(6) | 26.74 ± 1.07(6) | LSD | 0.032* |
|  |  | 27.12 ± 3.14(6) | / | 26.74 ± 1.07(6) | LSD | 1 |

Data are presented as mean ± standard deviation; n=3 dams/group (2 embryos/litter); CP, Cortical plate; df, Degrees of freedom; IZ, Intermediate zone; PGDM, Pregestational diabetes mellitus; SVZ, Subventricular zone; VZ, Ventricular zone; SD, Standard deviation; ****p<*0.001, ***p<*0.01 and **p<*0.05. For comparisons involving multiple groups, one-way analysis of variance (ANOVA) was employed, followed by the Student–Newman–Keuls post hoc test.

**Table S11. List of qPCR primers used.**

| **Primer** | **Sequence (5′→3′)** |
| --- | --- |
| Hes1 Forward | ATAGCTCCCGGCATTCCAAGCT |
| Hes1 Reverse | CGGTGTTAACGCCCTCACACGT |
| Hes5 Forward | AAACACAGCAAAGCCTTCGCCG |
| Hes5 Reverse | CAGGGTCAGGAACTGTACCGCC |
| Hey1 Forward | GCTGTGTTCCATGTCCCCAACG |
| Hey1 Reverse | TCCCGAAACCCCAAACTCCGAT |
| Hey2 Forward | CCAGGCTACAGGGGGTAAAGG |
| Hey2 Reverse | GGCACAGGTGCTGAGATGAGA |
| Dll1 Forward | GGGAGTGCAAGTGCAGAGTT |
| Dll1 Reverse | CACAGTGGTTGAAGGGGTTG |
| Notch1 Forward | CGGGACATCGCACAGGAGTA |
| Notch1 Reverse | CGTAGAGCTTGGGCTGCAC |
| Foxp1 Forward | CACCTCAGGTTATCACTCCTCA |
| Foxp1 Reverse | AGCTGCAACTGTTCCTGTTGT |
| Neurog2 Forward | AACTCCACGTCCCCATACAG |
| Neurog2 Reverse | GAGGCGCATAACGATGCTTC |
| Nr2f1 Forward | GCACTACGGCCAATTCACCT |
| Nr2f1 Reverse | TTGGAGGCATTCTTCCTCGC |
| Tubb3 Forward | TAGACCCCAGCGGCAACTAT |
| Tubb3 Reverse | GTTCCAGGTTCCAAGTCCACC |
| Reln Forward | CAACCCCACCTACTACGTTCC |
| Reln Reverse | TCACCAGCAAGCCGTCAAAAA |
| Dab1 Forward | GTGCTGTGACCCAATTAGAACT |
| Dab1 Reverse | GACGGGAGAAAGGCATCACC |
| Robo1 Forward | CCTTCAGACCTGATCGTCTCC |
| Robo1 Reverse | TGAGCGCGGGTCATCTTTG |

**Table S12. shRNA target sequences used in this study.**

| **shRNA name** | **Target sequence (5′→3′)** |
| --- | --- |
| shControl | ACTACCGTTGTTATAGGTGTT |
| shFoxp1-1 | GTGCGAGTAGAGAACGTTAAA |
| shFoxp1-2 | GCTTACCTCATACTCCAACAA |
| shFoxp1-3 | CGGAAGTTAGACCACCATTTA |
| shFoxp1-4 | GCTAACACTAAACGAAATCTA |
| shDll1-1 | TGGAGGACGATGTTCAGATAA |
| shDll1-2 | GAGCATGCACCCTGCCATAAT |
| shDll1-3 | AGCCGAGAAGAGCAGCTTTAA |
| shDll1-4 | CCTCACTGTGGGAGAAGAATG |
